# NOTCH target gene *HES5* mediates oncogenic and tumor suppressive functions in hepatocarcinogenesis

**DOI:** 10.1038/s41388-020-1198-3

**Published:** 2020-02-13

**Authors:** Sarah Luiken, Angelika Fraas, Matthias Bieg, Raisatun Sugiyanto, Benjamin Goeppert, Stephan Singer, Carolin Ploeger, Gregor Warsow, Jens U. Marquardt, Carsten Sticht, Carolina De La Torre, Stefan Pusch, Arianeb Mehrabi, Norbert Gretz, Matthias Schlesner, Roland Eils, Peter Schirmacher, Thomas Longerich, Stephanie Roessler

**Affiliations:** 1 Institute of Pathology, University Hospital Heidelberg, Heidelberg, Germany; 2 Center for Digital Health, Berlin Institute of Health and Charité – Universitätsmedizin Berlin, Berlin, Germany; 3 Heidelberg Center for Personalized Oncology (DKFZ-HIPO), Heidelberg, Germany; 4 German Cancer Research Center (DKFZ), Bioinformatics and Omics Data Analytics, Heidelberg, Germany; 5 First Department of Medicine, University Medical Centre of the Johannes Gutenberg University Mainz, Mainz, Germany; 6 Medical Research Centre, University of Heidelberg, Mannheim, Germany; 7 Department of Neuropathology, University Hospital Heidelberg, Heidelberg, Germany; 8 Clinical Cooperation Unit Neuropathology, German Cancer Research Center, Heidelberg, Germany; 9 Department of General Visceral and Transplantation Surgery, University Hospital Heidelberg, Heidelberg, Germany; 10 Health Data Science Unit, University Hospital Heidelberg, Heidelberg, Germany; 11 Translational Lung Research Center Heidelberg (TLRC), German Center for Lung Research (DZL), University of Heidelberg, Heidelberg, Germany

**Keywords:** Cancer genetics, Cancer genetics, Oncogenes

## Abstract

NOTCH receptor signaling plays a pivotal role in liver homeostasis and hepatocarcinogenesis. However, the role of NOTCH pathway mutations and the NOTCH target gene *HES5* in liver tumorigenesis are poorly understood. Here we performed whole-exome sequencing of 54 human HCC specimens and compared the prevalence of NOTCH pathway component mutations with the TCGA-LIHC cohort (*N* = 364). In addition, we functionally characterized the NOTCH target HES5 and the patient-derived HES5-R31G mutation in vitro and in an orthotopic mouse model applying different oncogenic backgrounds, to dissect the role of HES5 in different tumor subgroups in vivo. We identified nonsynonymous mutations in 14 immediate NOTCH pathway genes affecting 24.1% and 16.8% of HCC patients in the two independent cohorts, respectively. Among these, the HES5-R31G mutation was predicted in silico to have high biological relevance. Functional analyses in cell culture showed that HES5 reduced cell migration and clonogenicity. Further analyses revealed that the patient-derived HES5-R31G mutant protein was non-functional due to loss of DNA binding and greatly reduced nuclear localization. Furthermore, HES5 exhibited a negative feedback loop by directly inhibiting the NOTCH target HES1 and downregulated the pro-proliferative MYC targets ODC1 and LDHA. Interestingly, HES5 inhibited MYC-dependent hepatocarcinogenesis, whereas it promoted AKT-dependent liver tumor formation and stem cell features in a murine model. Thus, NOTCH pathway component mutations are commonly observed in HCC. Furthermore, the NOTCH target gene *HES5* has both pro- and anti-tumorigenic functions in liver cancer proposing a driver gene dependency and it promotes tumorigenesis with its interaction partner AKT.

## Introduction

In recent years, large next-generation sequencing projects sought to identify functionally important mutations, which may allow for the development of targeted drugs. These studies revealed cancer driver mutations, disease subtyping, tumor heterogeneity, downstream functional associations, and targeted precision therapies [1, 2]. However, in multiple tumor entities, including hepatocellular carcinoma (HCC), which is the most frequent form of liver cancer, only few genes are recurrently mutated [3–5]. For example, in The Cancer Genome Atlas Liver Hepatocellular Carcinoma (TCGA-LIHC) HCC data set (*N* = 364), only six genes were found to be significantly mutated in >5% of patients, namely *TP53*, *CTNNB1*, *ALB*, *AXIN1*, *ARID1A*, and *APOB* [3]. Even after including copy number alterations, a subgroup of HCC patients did not exhibit any genomic alteration, suggesting mutations in non-coding regions or epigenetic mechanisms of tumorigenesis as driving events. Consistent with the high heterogeneity of mutations, most mutations do not occur in hotspot regions. As mutations of different genes within the same pathway may result in the same outcome, combining multiple genes of one pathway is a powerful approach to identify significantly altered pathways [4, 5].

The NOTCH pathway is highly conserved and is a key factor of cell-fate determination, embryonic development, and adult tissue homeostasis [6]. In humans, four NOTCH receptors, NOTCH1–4, exist, which are temporarily and spatially differentially expressed. After NOTCH ligand binding, the NOTCH receptor undergoes a successive proteolytic cleavage cascade leading to the release of the NOTCH intracellular domain (NICD), which translocates to the nucleus. NICD binds to the transcription factor CBF1 (also known as CSL or RBP-Jκ) and acts as a transcriptional co-activator, ultimately resulting in the induction of NOTCH target gene expression [6]. Among the most common target genes exerting the canonical NOTCH pathway response are members of the *HES* (hairy and enhancer of split) family transcription repressors [7]. In the liver, the NOTCH pathway plays a pivotal role during liver development and regeneration processes controlling cell-fate decisions of bipotent liver progenitor cells promoting intrahepatic bile duct formation [8].

Persistent deregulation of NOTCH signaling also exerts a critical influence on liver inflammation, tumor development, and progression [9]. Albeit until now, published data with regard to the function of the NOTCH pathway in HCC are controversial [10]. On one hand, mice constitutively overexpressing NOTCH1 intracellular domain (N1ICD) in liver epithelial cells develop liver tumors resembling human HCC, suggesting an oncogenic function [11]. On the other hand, a tumor suppressive role was illustrated in mice with liver-specific inactivation of the Retinoblastoma (Rb) pathway, where overexpression of N1ICD inhibited cell growth and induced apoptosis [12]. These contradictory results suggest a context dependency of the NOTCH pathway readout and a close interaction with other signaling pathways. Most studies propose a tumor-promoting character of NOTCH, found NOTCH family receptors to be overexpressed in human HCC samples, and partially associated their expression with poor prognosis [13, 14]. Furthermore, 30% of HCC patients harbor tumor-associated hyper-activated NOTCH signaling [11] and NOTCH1 activation was increased in more aggressive HCC [15]. In mice, activated NOTCH2 signaling lead to HCC formation [16]. In addition, activated NOTCH1 together with AKT signaling resulted in the formation of intrahepatic cholangiocarcinoma (iCCA), which is the second most prevalent type of liver cancer [17, 18]. Recently, Fu et al. [19] reported that dual blockade of EGFR/PI3K/AKT and NOTCH signaling has the potential to decrease resistance and thus may gain clinical efficacy in triple-negative breast cancer.

In an effort to characterize the mutational landscape of HCC, whole-exome sequencing of 54 human HCC samples was performed (Heidelberg Center for Personalized Oncology, HIPO-HCC). We identified a considerable number of HCC samples carrying mutations in NOTCH signaling components. Among these was a single-base mutation in the NOTCH target gene *HES5* converting arginine 31 to glycine (R31G).

Considering that the majority of recent publications focused on the overall expression of *NOTCH* genes in human tissues or the modulation of NOTCH1 activity in mouse experiments, a better understanding of NOTCH pathway components such as HES5 is an important step towards understanding the precise function of the NOTCH downstream signaling cascade and for the development of targeted therapies. Thus, new insights into NOTCH signaling and interaction with other pathways in liver carcinogenesis are needed.

Here we functionally and biochemically analyzed the NOTCH target gene *HES5*, which is understudied. We were able to demonstrate that HES5 is a key regulator of NOTCH downstream signaling in liver carcinogenesis and exhibits context-dependent oncogenic and tumor suppressive features. In addition, we demonstrated that the patient-derived HES5-R31G mutation is non-functional, suggesting a tumor suppressive role in the affected HCC patient.

## Results

### Whole-exome sequencing reveals mutations and copy number alterations of NOTCH pathway components in HCC

**Fig. 1:**
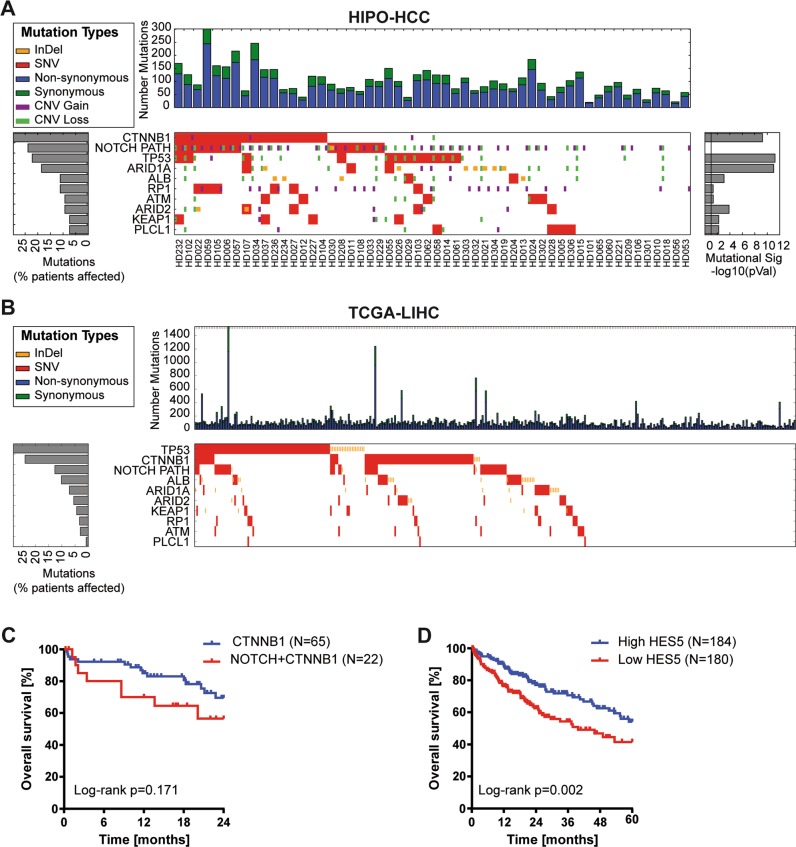
The NOTCH pathway is frequently altered in HCC. **a** Whole-exome sequencing reveals a high frequency of NOTCH pathway mutations in HCC patients of the HIPO cohort (*N* = 54). The nine most commonly altered genes and aberrations in NOTCH pathway genes, which had more than 5% frequency, are displayed. **b** Analysis of whole-exome sequencing data of the TCGA-LIHC cohort (*N* = 364) confirms a high frequency of NOTCH pathway mutations in HCC patients. **c** Kaplan–Meier curve showing overall survival of TCGA-LIHC cohort patients with *CTNNB1* mutation without NOTCH pathway mutation compared with patients with both *CTNNB1* mutation and NOTCH pathway mutation (log-rank test). **d** Kaplan–Meier curve showing overall survival of TCGA-LIHC cohort patients with HES5 mRNA expression above median (high HES5 group) compared with patients with HES5 mRNA expression below median (low HES5 group; log-rank test).

Previous reports employing next-generation sequencing analyses of HCC tissue samples showed that only few genes are recurrently mutated [4, 20–22]. Instead, most mutations occur at a very low frequency and, thus, it is difficult to pinpoint the relevance of these mutations in tumorigenesis. Here we performed whole-exome sequencing of 54 HCC patients from Heidelberg University Hospital (HIPO-HCC cohort, *N* = 54; Supplementary Table S1). Besides known frequent mutations in *CTNNB1* (29.6%), *TP53* (22.2%), and *ARID1A* (18.5%), we observed a variety of mutations in NOTCH pathway components (Supplementary Table S2 and Fig. 1a). In total, 19 mutations in 14 different genes affecting 24.1% (13/54) of patients in our cohort were identified in the NOTCH pathway (Fig. 1a and Supplementary Tables S1 and S3). All mutations were shown to be somatic via Sanger sequencing of the tumor and adjacent non-tumor tissue. In addition, copy number analysis revealed profiles similar to previous publications [3, 21, 23]. We observed DNA amplifications in the NOTCH pathway genes *APH1A*, *NCSTN*, *PSEN2*, *MAML1*, *NOTCH4*, *PTCRA*, and *DTX2*; however, it appeared that only few NOTCH pathway genes were affected by copy number alteration and no focal amplifications were observed (Supplementary Fig. S1A). A very similar picture was obtained, analyzing mutation (*N* = 364) and copy number data (*N* = 384) of the TCGA-LIHC cohort [3]. Multiple mutations in the NOTCH pathway but rare copy number alterations were observed in the TCGA-LIHC cohort (Fig. 1b, Supplementary Fig. S1B, and Supplementary Table S4). In the TCGA-LIHC cohort, a total of 78 mutations affecting at least one NOTCH pathway component were identified in 61 out of 364 (16.8%) patients.

Interestingly, NOTCH pathway mutations were significantly associated with co-occurrence of *CTNNB1* mutations in both cohorts (HIPO: *p* = 0.040; TCGA-LIHC: *p* = 0.009; Fisher’s exact test). Patients with NOTCH pathway and *CTNNB1* mutation had the trend of a poorer outcome compared with patients with *CTNNB1* but without NOTCH pathway mutations (Fig. 1c). In contrast, *TP53* mutation and *MYC* amplification were not associated with NOTCH pathway mutations (Fisher’s exact test *p* > 0.05). Thus, multiple NOTCH pathway components exhibited low-frequency mutations affecting a subset of HCC patients and NOTCH pathway mutations appear to be associated with *CTNNB1* mutations in a considerable number of HCC patients.

To pinpoint the NOTCH pathway mutations with the highest biological relevance, we applied six different algorithms to predict the functional impact and relevance to carcinogenesis of the NOTCH pathway mutations (Supplementary Table S3). Due to consistently high-impact prediction and because of its function as an integrative downstream transcription factor, we decided to functionally characterize HES5 wild-type (HES5wt) and HES5-R31G mutant (hereafter named HES5mut) protein in HCC. In addition, patients with low HES5 expression exhibited a significantly lower overall survival, suggesting a tumor suppressive function in at least a subset of HCC patients (Fig. 1d).

### HES5 expression is induced by NOTCH signaling in HCC cells

We tested whether HES5 might be activated by NOTCH signaling in liver cancer cell lines. Therefore, we screened liver cancer cell lines for abundance of the canonical N1ICD and predominantly non-canonical N3ICD (Supplementary Fig. S2A). Transient overexpression of N1ICD and N3ICD efficiently induced endogenous HES5 mRNA expression (Supplementary Fig. S2B–D). For further in vitro analyses, we chose Hep3B and SNU475, which show intermediate N1ICD, N3ICD and HES1, and low HES5 expression, and generated inducible N1ICD or N3ICD cells (Supplementary Fig. S2A). Uninfected cell lines served as control. In particluar, N1ICD expression significantly increased HES1 and HES5 mRNA and protein abundance in both cell lines (Supplementary Fig. S3). Thereby, N1ICD had a greater effect on HES5 mRNA levels compared with N3ICD reflecting canonical activation capacity and the HES5 protein level only increased upon N1ICD expression. The induction of HES5 was stronger compared with commonly referred NOTCH target HES1 possibly due to higher endogenous HES1 levels. The activation of HES5 protein expression already started 8 h after N1ICD induction and peaked at 24 h post induction (Fig. S3D). Thus, HES5 is strongly upregulated by N1ICD in human HCC cells.

### HES5wt but not HES5 mut reduces tumorigenic properties of HCC cells

**Fig. 2:**
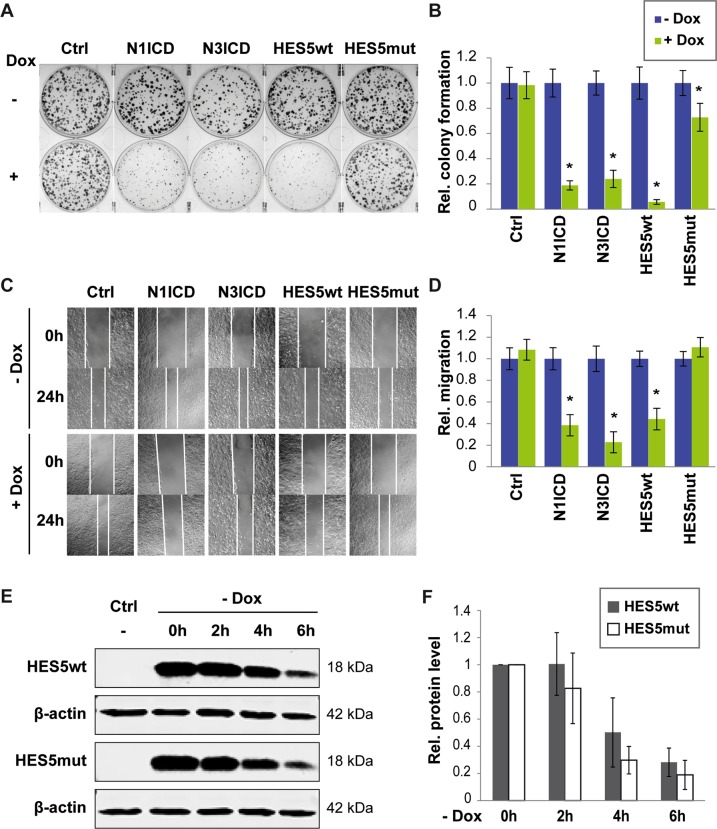
HES5wt but not HES5mut reduces colony formation and cell migration. **a** Representative images of colony formation assays of uninfected Hep3B (Ctrl) or Hep3B-expressing N1ICD, N3ICD, HES5wt, or HES5mut upon induction with 2 µg/ml Dox and (**b**) quantification of relative colony numbers of four independent experiments. **c** Representative images of scratch migration assays of uninfected Hep3B (Ctrl) or Hep3B-expressing N1ICD, N3ICD, HES5wt, or HES5mut upon treatment with 2 µg/ml Dox and (**d**) quantification of relative migration of four independent experiments. Data represent averages ± SD, *N* = 4. **e** HES5 wild-type (HES5wt) and HES5-R31G mutant (HES5mut) protein exhibit similar stability over time as determined by western blot analysis. Hep3B cells were treated with 2 µg/ml Dox for 24 h, followed by Dox withdrawal for indicated time points. Representative images are shown and β-actin served as loading control. **f** Quantification of HES5wt and HES5mut at 0, 2, 4, and 6 h after Dox depletion (*N* = 3). Half-life (*t*1/2) of HES5wt and HES5mut were 4.0 h and 2.3 h, respectively. *Mann–Whitney *U*-test *p* < 0.05.

To study the cell biological function of HES5wt and HES5-R31G (HES5mut) compared with N1ICD and N3ICD, inducible cell lines were generated. Colony formation and scratch migration assays showed that N1ICD, N3ICD, and HES5wt reduced single-cell clonogenicity and cell migration in Hep3B and SNU475 cells to a similar extent, whereas HES5mut had strongly reduced effects (Fig. 2a–d and Supplementary Fig. S4A, B). Cell viability was slightly reduced by N1ICD, N3ICD, and HES5wt in Hep3B but not in SNU475 cells (Supplementary Fig. S4C, D). In line with cell viability, we also observed that HES5wt reinforces cellular senescence in Hep3B cells as illustrated by positive staining of the senescence marker β-galactosidase (Supplementary Fig. S5) [24]. Furthermore, HES5mut protein half-life was slightly but not significantly reduced, confirming that HES5wt and HES5mut have similar expression levels and lower protein levels do not account for the loss-of-function (Fig. 2e, f). Thus, HES5wt inhibited tumor cell growth, migration, and clonogenicity, and promoted cellular senescence in vitro, whereas HES5mut appeared to have a loss-of-function phenotype.

### HES5 directly exhibits a negative feedback loop and suppresses the MYC target genes *ODC1* and *LDHA*

**Fig. 3:**
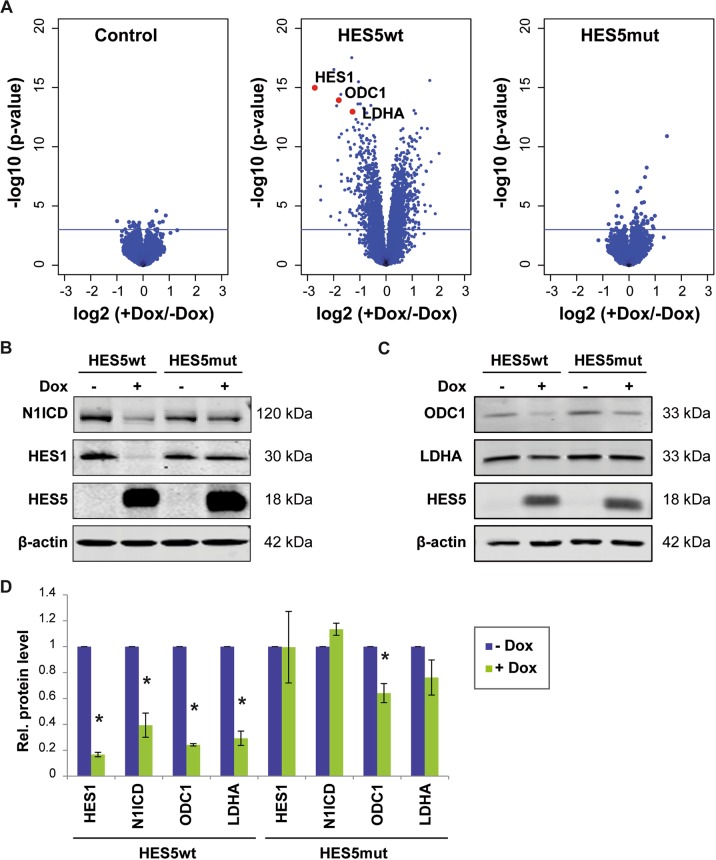
HES5wt but not HES5mut mediates extensive transcriptional repression. **a** Volcano plots showing the gene expression profiles of control (left), HES5wt-inducible (middle), or HES5mut-inducible (right) Hep3B cell lines with or without 2 µg/ml Dox treatment. The *x*-axis specifies the log2 fold changes and the *y*-axis specifies the negative log10 of the limma *t*-test *p*-values. The horizontal line indicates *p* = 0.001. Among genes with the highest negative fold change in HES5wt-expressing cells are *HES1*, *ODC1*, and *LDHA*, which are depicted by red dots in the middle panel. Accordingly, protein levels of HES1 and N1ICD (**b**) or ODC1 and LDHA (**c**) were analyzed by western blotting upon HES5wt or HES5mut expression in Hep3B cells. One representative experiment out of three with similar outcome is shown and β-actin served as loading control. **d** Quantification of protein levels of HES1, NOTCH1, ODC1, and LDHA in Hep3B cells following HES5wt or HES5mut induction obtained from three independent western blotting experiments. *Mann–Whitney *U*-test *p* < 0.05.

To identify downstream target genes of the transcriptional repressor HES5 and to compare the downstream signaling effects of HES5wt and HES5mut, we performed gene expression microarray analysis of Hep3B cells expressing HES5wt, HES5mut, or uninfected control cells with or without induction by doxycycline (Dox) treatment. Class comparison analysis revealed that Dox treatment of control, HES5wt, or HES5mut cells resulted in the differential expression of 27 genes, 2162 genes, or 55 genes, respectively (*p* < 0.001; with an adj. *p* = 0.05; Fig. 3a). Pathway analysis of the HES5wt target genes showed that enriched pathways involved stem cell-related pluripotency, PI3K-Akt signaling, and other cancer-related pathways (Supplementary Table S5). We noted that the gene downregulated with the highest fold change was *HES1* (Fig. 3a). NOTCH1 was also significantly downregulated through HES5wt. Thus, we validated in independent experiments that HES5wt but not HES5mut downregulated HES1 and NOTCH1 at the mRNA and protein level, respectively (Fig. 3b, d and Supplementary Fig. S6). Furthermore, we noticed that multiple HES5 target genes are known MYC targets. To validate this notion, we analyzed publically available MYC-ChIPseq data of HepG2 liver cancer cells from ENCODE (ENCSR000DLR) and found that of the 3509 genes with MYC-binding sites, 512 genes were deregulated by HES5wt (Supplementary Fig. S7 and Supplementary Table S6). Among these, HES5-regulated genes with the highest difference were the well-known MYC targets *ODC1* and *LDHA*, which constitute metabolic genes essential for rapid cell proliferation (Fig. 3a) [25–29]. Consistently, we found that ODC1 and LDHA were significantly downregulated at the protein level by HES5 (Fig. 3c, d).

**Fig. 4:**
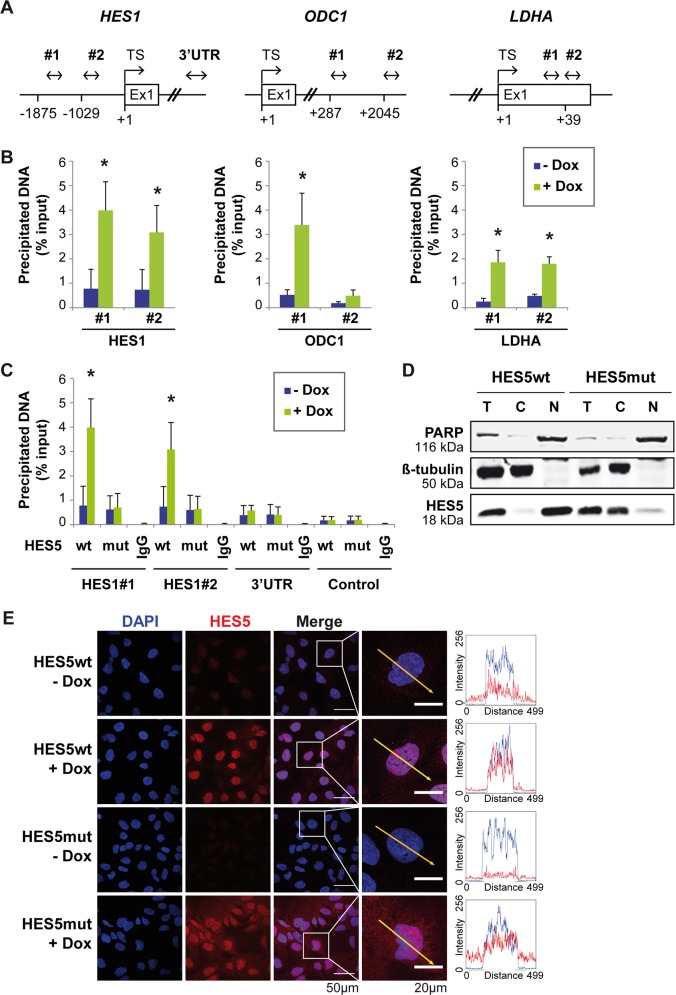
HES5wt directly binds *HES1*, *ODC1*, and *LDHA* genes, whereas HES5mut accumulates in the cytoplasm. **a** Schematic overview of *HES1*, *ODC1*, and *LDHA* genomic loci illustrates predicted HES5-binding sites and the position of PCR primers used for chromatin immunoprecipitation (ChIP). Proposed HES5-binding sites were located at position −1875 and −1029 in the *HES1* promoter (both 5′-CACGTG-3′, C-site/E-box), at position +287 (5′-CACGTG-3′, C-site/E-box) and +2045 (5′-CACATG-3′, E-box) in intron 1 of *ODC1*, and at position +39 in exon 1 of *LDHA* (5′-CACGCG-3′, C-site). **b** Binding of HES5wt to indicated sites of *HES1* (*N* = 3), *ODC1* (*N* = 4), and *LDHA* (*N* = 4) genes was verified by HES5-directed ChIP. **c** Furthermore, ChIP with HES5 antibody confirmed binding of HES5wt but not HES5mut protein to the analyzed consensus sequences in the *HES1* promoter upon induction with 2 µg/ml Dox treatment (*N* = 3). Negative control primers flank a region of genomic DNA between the *GAPDH* gene and the chromosome condensation-related SMC-associated protein (*CNAP1*) gene, which is deficient of transcription factor binding sites. Data represent averages ± SD. *Mann–Whitney *U*-test *p* < 0.05; TS: transcription start; 3′-UTR: 3′-untranslated region. **d** Western blotting of subcellular fractions of HES5wt- or HES5mut-expressing Hep3B cells. One representative experiment out of three with similar outcome is shown. PARP and β-tubulin serve as markers for the nuclear and cytoplasmic fraction, respectively. C: cytoplasmic fraction; N: nuclear fraction; T: total cell lysate. **e** Representative images of immunofluorescence in HES5wt- or HES5mut-expressing Hep3B cells with or without 2 µg/ml Dox treatment (*N* = 3). The yellow arrow (50 µm long) in the magnified image represents intensity profiles of HES5 (red signal) and DAPI (blue signal).

As aforementioned genes exhibited a pronounced downregulation by HES5, we aimed to study whether HES5 directly regulated their expression. In addition, we sought to test whether the loss-of-function of HES5mut may be caused by reduced binding affinity of HES5mut to genomic DNA. In silico analyses revealed potential HES5-binding motifs in the *HES1* promoter, intron 1 of *ODC1* and exon 1 of *LDHA* (Fig. 4a). Indeed, chromatin immunoprecipitation showed that HES5wt differentially bound to predicted binding sites of the respective genomic loci (Fig. 4b). In contrast to HES5wt, HES5mut did not bind to the *HES1* promoter in chromatin immunoprecipitation (ChIP) experiments (Fig. 4c). Genomic regions within the 3′-untranslated region of *HES1* and between *GAPDH* and *CNAP1*A served as negative controls (Fig. 4c). Thus, HES5wt directly bound to the *HES1*, *ODC1*, and *LDHA* genes resulting in transcriptional repression but HES5mut failed to efficiently regulate gene expression.

### HES5mut fails to translocate to the nucleus

The reduced binding of HES5mut to the *HES1* promoter might be accompanied by altered HES5mut protein localization. To test this hypothesis, we performed cell fractionation experiments followed by western blot analyses of HES5. HES5wt protein was mainly located in the nucleus, whereas HES5mut was predominantly detected in the cytoplasmic fraction (Fig. 4d). In addition, immunofluorescence of Hep3B and SNU475 cells confirmed that HES5mut localized preferentially to the cytoplasm compared with HES5wt (Fig. 4e and Supplementary Fig. S8A). As nuclear shuttling of small proteins such as HES5 has been shown to be often mediated by nuclear pore proteins, we knocked down several nuclear pore components to test whether HES5 localization is affected. Herein, we depleted the central core proteins NUP62 and NUP98, and the nuclear pore basket protein NUP153. However, none of them had an effect on nuclear import of HES5 (Supplementary Fig. S8B). Thus, reduced nuclear translocation of HES5mut is probably independent of active nuclear pore transport.

### AKT directly binds HES5 protein

**Fig. 5:**
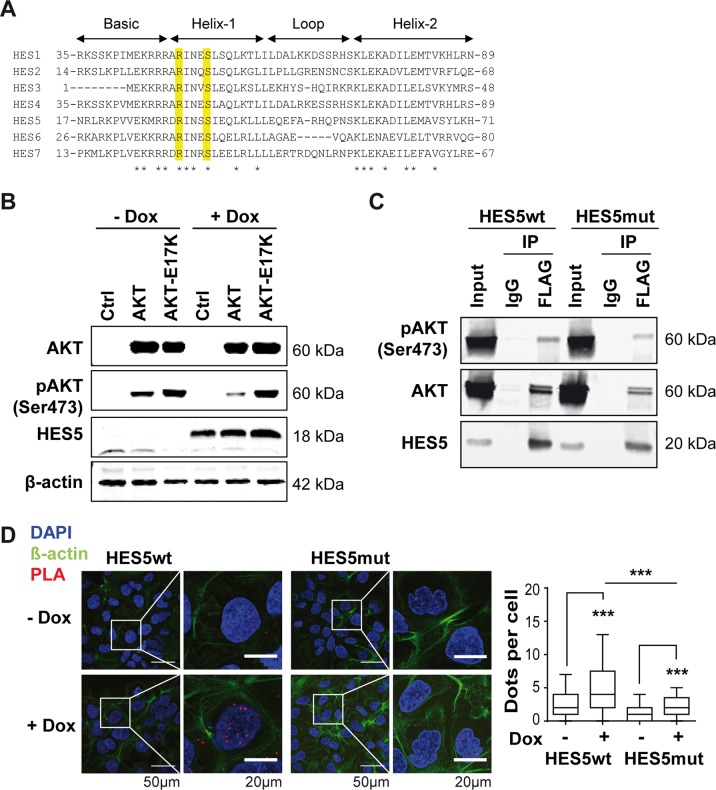
HES5 directly interacts with AKT. **a** Alignment of the N-terminal basic helix-loop-helix domain of human HES1–7 proteins. The conserved arginine (R) and serine (S) residues of a potential AKT phosphorylation motif are highlighted in yellow. Asterisks denote amino acids conserved in all seven HES family members. **b** Western blotting of HES5-inducible Hep3B cells transfected with empty vector control (Ctrl), AKT, or hyperactive mutant AKT-E17K and treated with 2 µg/ml Dox or left untreated. One representative experiment out of three with similar outcome is shown and β-actin served as loading control. **c** Co-immunoprecipitation experiments of HEK293T cells transiently co-transfected with HES5wt-FLAG (left) or HES5mut-FLAG (right) together with AKT. After anti-FLAG immunoprecipitation, samples were separated by SDS–PAGE and subjected to western blot analysis with anti-pAKT(Ser473), anti-AKT, or anti-HES5-reactive antibodies. One representative experiment out of three with similar outcome is shown. **d** Representative images of proximity ligation assays (PLAs) in Hep3B cells expressing HES5wt or HES5mut protein with or without induction by 2 µg/ml Dox treatment (left panel). Quantitative representation of PLA dots per cell by boxplots with whiskers representing 5–95% confidence intervals (right panel). *N* = 93–113 cells of 8 images analyzed for each condition; ***Mann–Whitney *U*-test *p* < 0.001.

We analyzed the amino acid sequence and domains of HES5 for potential functions of the HES5-R31G mutation. HES5 harbors three functionally important regions: the basic helix-loop-helix (bHLH), the Orange and WRPW domains in the N- to C-terminal direction [30]. Alignment of the seven HES family members HES1–7 revealed that HES5-arginine 31 is located in helix-1 of the N-terminal bHLH and highly conserved (Fig. 5a). Screening for motifs of protein interaction and modification suggested S34 and/or S35 to be potentially phosphorylated by AKT. Furthermore, S35 is highly conserved within the HES protein family (Fig. 5a). As AKT has been demonstrated to function as an oncogene in HCC, we analyzed the influence of AKT on HES5 protein and tested whether AKT may directly interact with HES5. Overexpression of AKT or the hyperactive variant AKT-E17K did not significantly increase HES5wt protein levels (Fig. 5b). To examine whether AKT directly binds HES5, we performed co-immunoprecipitation (co-IP) and proximity ligation assays (PLAs). HES5wt-FLAG or HES5mut-FLAG protein was pulled down and bound AKT protein was detected by immunoblot showing that both HES5wt and HES5mut directly bound total AKT and Ser473-phosphorylated active AKT (Fig. 5c). Confocal imaging of PLA demonstrated that HES5wt directly interacted with AKT but HES5mut exhibited significantly reduced interaction (Fig. 5d). In contrast, HES1-FLAG protein only very weakly bound AKT protein in co-IP experiments, suggesting that the AKT-HES5-interaction may not be a general function of HES family proteins (Fig. S9). Thus, AKT directly interacted with HES5wt in vitro and in situ.

Next, we analyzed phosphomimetics of the potential AKT phosphorylation sites S34 and S35, to determine the effect of HES5 phosphorylation on protein function and localization. Interestingly, the phosphomimetic HES5-S35D but not HES5-S34D displayed a loss-of-function phenotype similar to HES5mut in the colony formation assay (Supplementary Fig. S10A). In addition, HES5-S34D repressed HES1 protein abundance similar to HES5wt, whereas HES5-S35D did not (Supplementary Fig. S10B). Consistently, HES5-S35D but not HES5-S34D accumulated in the cytoplasm as shown by cell fractionation followed by western blotting and also by immunofluorescence imaging (Supplementary Fig. S10C, D). Even though phosphomimetics suggested a functional importance of S35, the validity of the phosphomimetic approach is limited and non-phospho-specific effects cannot be excluded. Thus, the effect of a potential phosphorylation of HES5 and the involvement of AKT in this process remains unresolved.

### HES5 suppresses MYC-dependent hepatocarcinogenesis

**Fig. 6:**
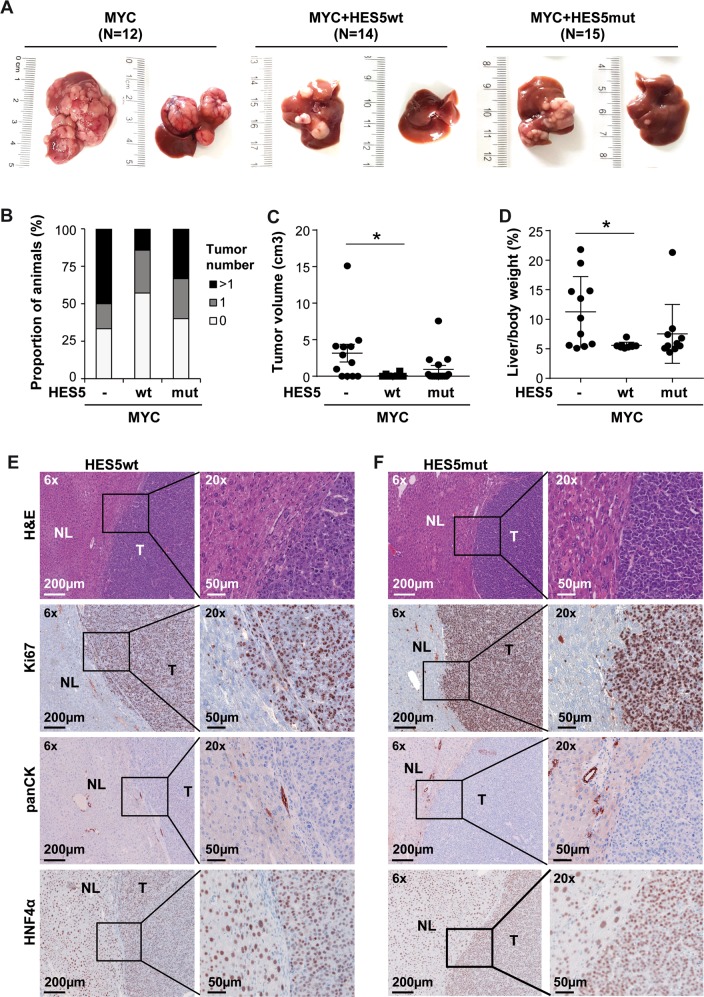
HES5wt but not HES5mut reduces MYC-induced hepatocarcinogenesis. **a** Representative images of murine livers after hydrodynamic injection of transposon vectors encoding MYC (*N* = 12), MYC/HES5wt (*N* = 14) or MYC/HES5mut (*N* = 15) into FVB/N mice. Macroscopically, livers of MYC mice appeared enlarged and displayed numerous nodules. Livers of male (left) and female mice (right) were collected 6 or 8 weeks after injection, respectively, and the number of macroscopically detectable tumors was determined. **b** Quantification of the number of nodules, **c** the tumor volume per liver, and **d** the liver to body weight ratio of FVB/N mice. *Mann–Whitney *U*-test *p* < 0.05. **e**, **f** Representative H&E, anti-Ki67, anti-panCK, and anti-HNF4α staining of liver tissue of MYC/HES5wt (**e**) or MYC/HES5mut (**f**) transduced murine livers. H&E, hematoxylin and eosin; NL, normal liver; T, tumor.

To explore the function of HES5 in vivo, we induced liver tumors in mice using hydrodynamic tail vein injection with transposon vectors expressing MYC. Consistent with previous publications, MYC alone resulted in multiple large tumor nodules within 6–8 weeks after injection (Fig. 6a) [31]. Interestingly, HES5wt but not HES5mut decreased liver tumor formation in comparison with MYC alone (Fig. 6b–d). Tumors resulting from MYC/HES5wt or MYC/HES5mut transduction exhibited trabecular, solid growth patterns and were highly proliferative as evidenced by high numbers of Ki67-positive cells, negative for pan-CK (cytokeratin), and positive for hepatocyte nuclear factor 4α (HNF4α), suggesting hepatocellular differentiation (Fig. 6e, f). Consistent with anti-proliferative effects ascertained in Hep3B cells, Ki67 staining appeared reduced in MYC/HES5wt compared to MYC/HES5mut tumors. Thus, in vitro in HCC cell lines and in vivo in a MYC-driven HCC model, HES5 suppressed tumorigenic functions.

### HES5 accelerates AKT-mediated liver tumor formation

**Fig. 7:**
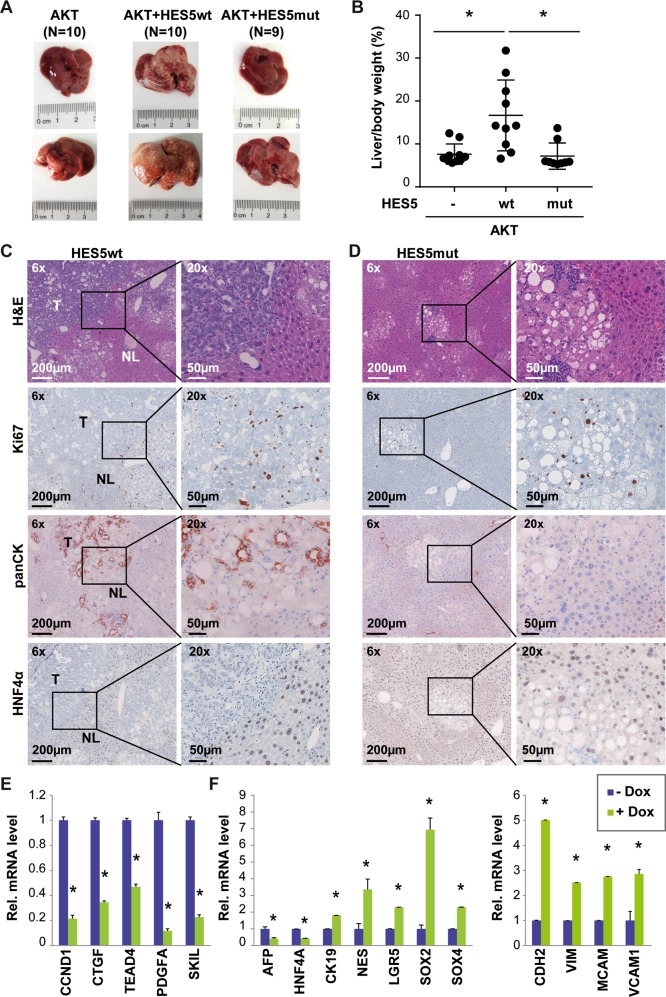
HES5wt but not HES5mut promotes AKT-induced liver tumorigenesis of a mixed HCC/iCCA phenotype in mice. **a** Representative images of male (top) or female (bottom) murine livers and **b** liver to body weight ratio of FVB/N mice 13 weeks after hydrodynamic injection with transposon vectors encoding myrAKT (*N* = 10), myrAKT/HES5wt (*N* = 10), or myrAKT/HES5mut (*N* = 9). **c**, **d** Representative H&E, anti-Ki67, anti-panCK, and anti-HNF4α immunohistochemical staining of tumors in myrAKT/HES5wt (**c**) or myrAKT/HES5mut (**d**) transduced murine livers. H&E, hematoxylin and eosin; NL, normal liver; T, tumor. (**e**) Expression of genes associated with proliferation or (**f**) differentiation/stem cell characteristics (left) and EMT (right) in HES5wt-inducible Hep3B cells with or without Dox treatment, as indicated (*N* = 3). *Mann–Whitney *U*-test *p* < 0.05.

As HES5 directly bound AKT and AKT together with active NOTCH signaling has been shown to increase tumorigenicity, we performed hydrodynamic transduction of hyperactive myristylated AKT (myrAKT) with or without HES5. MyrAKT alone resulted in a spotty pale appearance of murine livers without tumor formation within 13 weeks post injection, whereas hepatocarcinogenesis reportedly requires 28 weeks (Fig. 7a) [32]. When co-expressing HES5wt/myrAKT, livers were enlarged and multiple tumors were detectable at 13 weeks post injection. In contrast, HES5mut/myrAKT-transduced livers resembled myrAKT alone and did not show any macroscopic tumors (Fig. 7a). In addition, the liver to body weight ratio of HES5wt/myrAKT mice (*N* = 10) was significantly higher compared with myrAKT (*N* = 10) or HES5mut/myrAKT mice (*N* = 9; Fig. 7b). Histological analyses demonstrated that HES5wt/myrAKT induced liver tumors of mixed HCC/iCCA differentiation, whereas in HES5mut/myrAKT livers, clusters of clear-cell pre-neoplastic foci but no tumors were detected (Fig. 7c, d). Immunohistochemical staining showed heterogeneous pan-CK staining and low-to-absent HNF4α staining in HES5wt/myrAKT tumors, suggesting a mixed HCC/iCCA differentiation (Fig. 7c). In addition, we found that the genes *Vim*, *Mcam*, and *Ck19* were induced in HES5wt/myrAKT murine liver tumors (Supplementary Fig. S11). This mixed differentiation pattern in murine tumors was consistent with gene expression profiles obtained in the inducible Hep3B cell lines. Therein, proliferation-related factors such as CyclinD1 (CCND1), CTGF, TEA domain 4 (TEAD4), PDGFA, and SKIL were decreased (Fig. 7e). Although the Hippo/yes-associated protein (YAP) pathway components TEAD4 and CTGF were affected by HES5wt, other typical YAP-target genes and phospho-YAP protein were not generally altered (Supplementary Fig. S12). Furthermore, the hepatocyte-specific transcription factor HNF4A and AFP were decreased, whereas the cholangiocyte marker CK19 and the stem cell factors LGR5 and SOX4 were induced upon HES5wt expression (Fig. 7f). Also the epithelial-mesenchymal transition (EMT) markers CDH2, VIM, MCAM, and VCAM1 were significantly elevated, indicating enhanced EMT upon HES5 expression (Fig. 7f). Thus, in contrast to MYC-driven liver tumorigenesis, HES5 enhanced AKT-dependent tumorigenesis and induced a shift towards cholangiocyte differentiation.

## Discussion

Liver cancer is the second leading cause of cancer-related death worldwide, its incidence is rising and its mortality is increasing more rapidly than for other tumor entities [33, 34]. Thus, there is a great need for better understanding of the underlying molecular pathways and development of new therapeutic strategies based on these findings is required. Here we performed biochemical and functional analyses in vitro and in vivo to elucidate the function of the poorly understood NOTCH target gene *HES5* in liver carcinogenesis. Previous studies focused on the overexpression of hyperactive NICD in selected mouse models; albeit the role of downstream signaling transcription factors is still elusive.

**Fig. 8:**
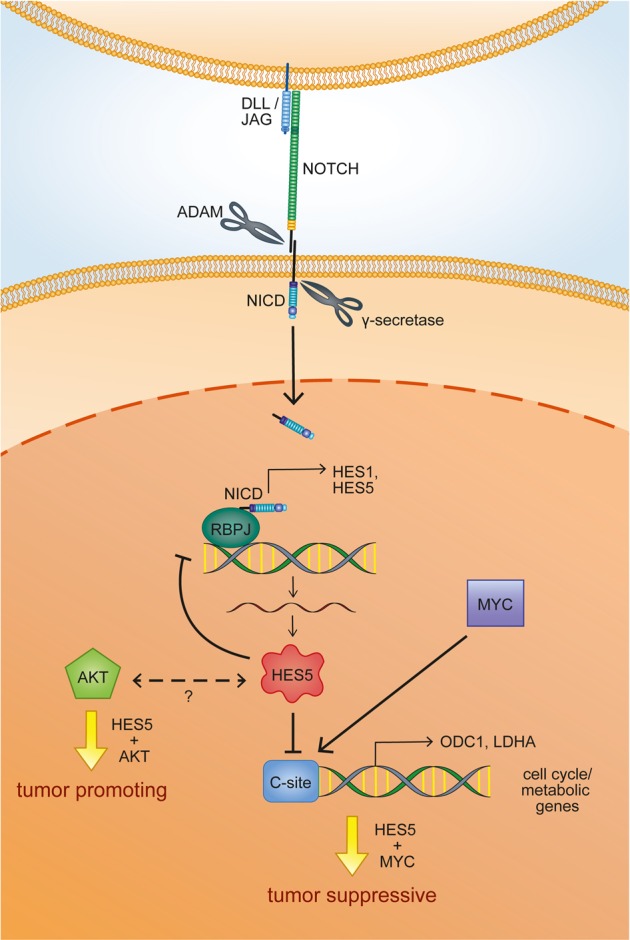
Schematic representation of HES5 cellular functions. Trans-cellular interaction of a Delta-like (DLL) or Jagged (JAG) family ligand to one of the four NOTCH receptors initiates a proteolytic cascade by an ADAM family protease and the γ-secretase complex, and results in the release of the NOTCH intracellular domain (NICD) into the cytoplasm of the NOTCH-expressing cell. The NICD is rapidly translocated into the nucleus, where it binds to the transcription factor RBPJ and activates the expression of downstream NOTCH target genes, among them the transcriptional repressors HES1 and HES5. HES5 promotes a negative NOTCH pathway circuitry inhibiting NOTCH1 and HES1 expression. Furthermore, it binds to C-site/E-box elements in cell cycle/metabolic genes such as *ODC1* and *LDHA*, which are also MYC target genes, thereby prohibiting their expression. Thus, the contradictory regulation of a subset of MYC target genes by HES5 might explain the tumor suppressive function in a MYC-driven tumor model. In contrast, HES5 directly binds to AKT and appears to synergistically promote AKT-dependent carcinogenesis in a so far unresolved manner.

To better understand the role of NOTCH signaling in HCC, we analyzed the function of the direct NOTCH target gene *HES5*. The rational for choosing HES5 was based on the predicted high impact of HES5-R31G on protein function. In HCC cell lines, we found that HES5 is highly induced by NOTCH signaling and the induction is even higher than of HES1. Furthermore, HES5 reduced cell migration and clonogenicity and induced cellular senescence similarly to N1ICD [35–37]. In addition, HES5 repressed genes associated with cell proliferation and hepatocyte differentiation, whereas progenitor cell and EMT-related genes were increased. In vivo, we could show that in MYC-induced liver tumors, HES5 repressed tumor growth, whereas in AKT-induced liver tumors, HES5 promoted tumor formation (Fig. 8).

Expression of hyperactive N1ICD together with AKT acts as a strong oncogene in the liver and promotes rapid iCCA development [18]. Consistently, overexpression of N1ICD in hepatocytes and biliary epithelium results in the induction of iCCA formation [38]. This suggested that activation of NOTCH signaling induced iCCA but not HCC. However, Abitbol et al. [39] recently showed that AXIN1 mutation in hepatocytes resulted in the development of HCC highly enriched in NOTCH and YAP oncogenic signatures. One may speculate that iCCA formation might result from hyperactive NOTCH signaling, whereas more physiological levels of endogenously activated NOTCH may favor HCC development. N1ICD also inhibited tumor cell growth and induced tumor cell apoptosis in mice with liver-specific inactivated Rb pathway, suggesting a tumor suppressive role of NOTCH in HCC [12]. Thus, the role of NOTCH signaling in HCC appeared controversial and might be provoked by the usage of different mouse models.

Here we demonstrated that HES5 inhibits MYC-driven HCC. This finding is also supported by the tumor suppressive effect of NOTCH in Rb-deficient hepatocytes as Rb has been demonstrated to induce binding of MYC to E-boxes within promoters of cell cycle-related genes leading to cell proliferation [12, 40]. Interestingly, HES5 inhibited gene expression of *HES1*, *ODC1*, and *LDHA*, and bound to their genomic loci containing C-site/E-box motifs. Thus, we showed that HES5 exhibited a negative feedback loop via HES1, similar to findings showing that HES proteins downregulate themselves resulting in oscillatory expression [7, 41]. Moreover, predicted HES5-binding sites were overlapping with published MYC-binding sites in *ODC1* and *LDHA* genes [27–29]. This inhibitory effect of HES5 binding to C-site/E-box elements of MYC targets, such as ODC1 and LDHA, may also explain its suppressive effect on MYC-driven tumorigenesis in mice, especially in regard to pro-proliferative genes (Fig. 8). Consistently, HES5 has been suggested to exhibit tumor suppressive effects in B-cell acute lymphoblastic leukemia and glioma [42, 43].

In contrast to MYC-induced tumors, AKT-induced liver tumor formation took considerably longer and led to a different morphology [32]. Interestingly, HES5 expression in HCC cells reduced HNF4α and increased CK19 levels, suggesting a transdifferentiation from HCC towards a progenitor/iCCA phenotype. In addition, the pro-proliferative genes *CCND1*, the yes-associated protein (YAP)-target gene *CTGF*, and the YAP interaction partner TEA domain 4 (*TEAD4*) were also reduced upon HES5 expression. This indicated that HES5 leads to a blockage of cell proliferation and induction of stem cell and EMT-gene induction. At the same time, stem cell-related genes, such as *NES*, *LGR5*, *SOX2*, and *SOX4* [44–46], and EMT-related genes were upregulated by HES5 induction. SOX2 is a main downstream regulator of SIRT1‐mediated self‐renewal and tumorigenic potential of liver cancer stem cells, and *SOX2* is amplified and drives proliferation in small-cell lung cancer [47, 48]. In addition, SOX4 induces *HES5* expression by binding to its promoter, thereby inhibiting differentiation of neural stem cells into oligodendrocytes [49]. This was also reflected by heterogeneous induction of the cholangiocyte marker pan-CK and inhibition of HNF4α in our AKT/HES5-liver cancer mouse model. Therefore, HES5 may lead to HCC cell de-differentiation and together with AKT displayed an oncogenic function (Fig. 8). However, AKT/HES5 murine tumors differed from AKT/N1ICD-induced murine tumors. First, AKT/N1ICD tumors developed within 3–4 weeks after hydrodynamic transduction, whereas AKT/HES5 tumors required 13 weeks to develop. Second, AKT/HES5 tumors exhibited stem cell-like mixed HCC/iCCA differentiation pattern and AKT/N1ICD tumors were iCCA [17, 18]. In support of the pro-tumorigenic effects of AKT and HES5, we found that both proteins directly bound each other. Hence, HES5 recapitulated partly the function of N1ICD and, in combination with AKT, HES5 acted as an oncogene.

Importantly, HES5-R31 and HES5-S35 are part of the HES5 bHLH domain, which is involved in DNA binding. Nuclear translocation of HES5 was independent of active transportation by the nuclear pore complex suggesting free shuttling of the 18 kDa HES5 protein. Therefore, loss of HES5 DNA binding may result in loss of nuclear retention and reduced levels of HES5 in the nucleus. Thus, these results suggested that HES5-R31G is a loss-of-function mutation and HES5 may act as a tumor suppressor. It will be interesting to further characterize HCC cases with regard to *CTNNB1* mutation status, which was significantly associated with NOTCH pathway mutations. Noteworthy, the HCC sample with HES5-R31G mutation of our study has an activating *CTNNB1* mutation but no *MYC* amplification. Activating *CTNNB1* mutations may favor inactivating NOTCH pathway mutations, as WNT and NOTCH signaling fulfill opposing roles in hepatic progenitor cells promoting differentiation into hepatocytes or cholangiocytes, respectively [50]. Further investigations will hopefully shed light on the WNT-NOTCH pathway interplay.

In the present study, we showed that mutations in NOTCH pathway components existed in 16.8–24.1% of HCC patients. NOTCH effectively induced HES5 expression and HES5 exhibited pro- and anti-tumorigenic effects in a cell-context or tumor-driver-dependent manner. We demonstrated that the HES5-R31G mutation is biologically relevant and leads to loss-of-function. As therapeutic inhibition of NOTCH signaling using β-secretase inhibitors, NOTCH receptor-blocking antibodies, or NOTCH transcription complex-blocking peptides is already tested in clinical trials, it is important to consider dual roles of NOTCH signaling [8]. The recent development of dual-targeting antibodies against EGFR/PI3K/AKT and NOTCH signaling is encouraging, because this dual inhibitor decreased resistance and thus may gain clinical efficacy in triple-negative breast cancer [19]. However, more detailed knowledge about NOTCH pathway signaling and cell type-dependent downstream effectors is required to design new effective drugs, avoiding harmful side effects and improving patient survival. It will be very interesting to analyze additional mutations of NOTCH pathway components to uncover the effect of NOTCH signaling in HCC.

## Materials and methods

### Patient samples and clinicopathological data

The study comprised 54 HCC patients (Supplementary Table S1) of whom fresh frozen tumor and paired non-tumor tissues were available. All tissue samples were provided by the Tissue Bank of the National Center for Tumor Diseases (NCT, Heidelberg, Germany) in accordance with the regulations of the NCT Tissue Bank. Informed consent in writing was obtained from each patient. The study protocol was approved by the ethics committee of Heidelberg University (S-206/2005, S-207/2005, and S-539/2012). Each HCC tumor sample was histologically confirmed by at least two experienced pathologists (BG, PS, TL).

### Cell lines

Eleven liver cancer cell lines (HuH1, HuH7, SNU182, SNU475, HepG2, Hep3B, HLE, HLF, PLC, KMCH1, and HUCCT1), the immortalized hepatocyte cell line HHT4 (provided by Curtis C. Harris), and HEK293T cells were used in this study. Cell lines were regularly tested for mycoplasma contamination (MycoAlert, Lonza, Basel, Switzerland), authenticated by short tandem repeat analysis and cultured as described previously [51, 52]. Briefly, HuH1, HuH7, HLF, HLE, PLC, KMCH1, and HEK293T cells were cultured in DMEM medium, Hep3B cells in MEM medium and HUCCT1, HepG2 and SNU182 in RPMI1640 medium supplemented with 10% fetal bovine serum (FBS; Thermo Fisher Scientific, Offenbach, Germany) and 1% Penicillin–streptomycin (100 IU/ml and 100 g/ml, respectively). SNU475 cells were grown in RPMI1640 medium containing 20% FBS and 1% Penicillin–streptomycin. All media and Penicillin–streptomycin were obtained from Sigma-Aldrich (Taufkirchen, Germany). Cell lines were transfected using Lipofectamine 2000 transfection reagent (Thermo Fisher Scientific) or polyethylenimine (Polysciences, Warrington, PA, USA) according to the manufacturer’s instructions.

### Statistical analysis

The statistical analysis of in vitro and mouse experiments was carried out using GraphPad Prism 6. Data are expressed as the mean ± SD. To compare differences between two groups, Student’s *t*-test was used. Fisher’s exact test was performed to test for association of mutation co-occurrence in patients sequenced. A *p*-value < 0.05 was considered significant.

## Supplementary information

SUPPLEMENTAL MATERIAL

SUPPLEMENTAL Table S4

## Data Availability

Whole-exome sequencing data were deposited in the European Genome-phenome Archive under accession EGAS00001003329. Gene expression microarray data of inducible Hep3B cell lines were deposited at the Gene Expression Omnibus (https://www.ncbi.nlm.nih.gov/geo/) under GSE121362.

## Code availability

All code used are available upon request. Additional Materials and Methods are available in the Supplementary Information including Supplementary Tables S7–S10.
