# Supplementary material for: NOTCH target gene HES5 mediates oncogenic and tumor suppressive functions in hepatocarcinogenesis
Source: Oncogene. 2020 Feb 13;39(15):3128–44. doi: 10.1038/s41388-020-1198-3 (PMC7142020; doi:10.1038/s41388-020-1198-3)
Supplement: Supplementary file 1 — SUPPLEMENTAL MATERIAL [file 41388_2020_1198_MOESM1_ESM.docx]

***Supplemental Information***

**NOTCH target gene HES5 mediates oncogenic and tumor suppressive functions in hepatocarcinogenesis**

Sarah Luiken, Angelika Fraas, Matthias Bieg, Raisatun Sugiyanto, Benjamin Goeppert, Stephan Singer, Carolin Ploeger, Gregor Warsow, Jens U. Marquardt, Carsten Sticht, Carolina De La Torre, Stefan Pusch, Arianeb Mehrabi, Norbert Gretz, Matthias Schlesner, Roland Eils, Peter Schirmacher, Thomas Longerich, Stephanie Roessler

**Supplemental Methods**

***Genomic DNA isolation and whole exome sequencing***

Genomic DNA was isolated from fresh frozen tissue using the QIAamp DNA micro kit (Qiagen, Hilden, Germany) for whole exome sequencing according to the manufacturer’s instruction. Genomic DNA was analyzed using a Qubit 2.0 Fluorometer (Thermo Fisher Scientific, Offenbach, Germany) and a 2100 Bioanalyzer system (Agilent, Santa Clara, CA, USA) for quality control and quantification. Whole exome sequencing libraries were prepared from DNA isolated from fresh frozen tissue and from macrodissected surrounding normal tissue to distinguish somatic from germline mutations. Exome capturing was performed using SureSelect Human All Exon V5+UTRs in-solution capture reagents (Agilent). Briefly, 1.5 μg genomic DNA were fragmented to 150–200 bp insert size with a Covaris S2 sonicator device (Woburn, MA, USA) and 250 ng of Illumina adapter-containing libraries were hybridized with exome baits at 65 °C for 16 h. Paired-end sequencing (2×101 bp) was carried out with a HiSeq 2500 instrument (Illumina, San Diego, CA, USA).

***Whole exome sequencing data analysis***

Whole exome sequencing reads were aligned against the 1000 genomes project’s phase 2 reference sequence including decoy sequences using bwa-aln (version 0.6.2) [1]. Subsequent duplicate marking was performed using the Picard software (https://broadinstitute.github.io/picard/).

For SNV calling we used our in-house mutation detection workflow that is based on samtools-mpileup [2-4]. This workflow was also used in the ICGC PCAWG project and can be accessed via the Dockstore webpage (https://dockstore.org/containers/quay.io/pancancer/pcawg-dkfz-workflow) [5]. In a first step, the workflow detected variants in the tumor sample and compared these to the matched control to distinguish between somatic and germline variants. Afterwards, the raw calls were annotated using several publicly available databases, like 1000 Genome variants, single nucleotide polymorphisms (dbSNP), repeats and other elements. Functional impact of SNVs was assessed using Annovar [6]. Raw InDel calls were detected using Platypus (version 0.7.1) [7]. Confidence assessment and annotation were done similar to SNV processing. Mutational significance – including SNVs and small InDels - of single genes was extracted from the MutsigCV results after running intogen (version 3.0.5) [8].

CNAs were inferred from whole exome sequencing data with cnvKit (version 0.9.0) with default parameter settings [9]. Heterozygous SNPs were determined as those positions with alternative allele fraction between 0.3 and 0.7 in the respective normal sample. Segments which contained at least 20 heterozygous SNPs were further processed to infer sample ploidy and tumor cell content along with allele-specific copy number estimates. The segments were classified as balanced or imbalanced according to the distribution of the frequencies of the alternative allele of the SNPs in the respective segments. If this distribution had a global maximum between 0.45–0.55 a segment was called balanced, remaining segments were further separated into two groups – ambiguous segments with one density peak outside of the above mentioned interval and imbalanced segments with two peaks. Ambiguous segments were neglected in subsequent steps. For imbalanced segments, the mean B-allele frequency (BAF) of all SNPs in the segment that were heterozygous in the germline was estimated using the allele with the higher read count as B-allele. Then, the mean read count of the B-allele was calculated as product of total coverage and the BAF of the respective segment. Tumor cell content (TCC) and ploidy of a sample were estimated using a method adapted from ACEseq [10]. For TCC estimation values in the range of 0.05–1.0 were tested, whereas a ploidy range between 1 and 6.5 was allowed. For each possible combination of TCC and ploidy, absolute copy numbers and allele-specific copy numbers and the decrease in heterozygosity (DH) were estimated segment-wise. Allele-specific copy numbers were calculated as total copy number divided by two for balanced segments and as a function of coverage and B-allele read counts in case of imbalanced segments. The weighted mean distance of all segments to the next allowed integer copy number state was calculated for total and allele-specific copy numbers. Here, “allowed” means even total copy number states for balanced segments, any integer copy number state for imbalanced segments and allele-specific copy numbers. TCC/ploidy combinations requiring negative copy number states or a DH larger than 1 for any segment was excluded. Local minima in the weighted mean distance were considered as possible TCC/ploidy solution for the sample and were visually evaluated.

As an independent source of validation for our findings, we used small somatic variants and copy number data of the TCGA-LIHC cohort (<https://portal.gdc.cancer.gov/projects/TCGA-LIHC>) [11]. We downloaded mutation data for 364 cases for which variant calls defined by mutect2 were available^37^. Furthermore, we downloaded copy number variation data for 384 cases for which CNV analysis was performed by DNAcopy (https://bioconductor.org/packages/release/bioc/html/DNAcopy.html). We defined segments that had a ploidy deviation of plus or minus 0.7 copy numbers from the tumor‘s ploidy as either being amplified or deleted and used these segments to assign copy number states to genes. This information was also used to create the genome-wide, cohort-wise copy number frequency plots.

For the analysis of genomic alterations in NOTCH pathway components, we included genes from the Gene Set Enrichment Analysis tool (KEGG PATHWAY hsa04330), except for ubiquitous transcription factors and chromatin modifying proteins (CIR1, CREBBP, CTBP1, CTBP2, EP300, HDAC1, HDAC2, KAT2A, KAT2B and NCOR2) and added the two recently identified NOTCH pathway Rabconnectin-3/DMXL2 and GXYLT1/2 regulators manually which lead to a total of 41 NOTCH pathway genes (Table S2) [12, 13].

To pinpoint the most relevant mutations, we applied six online tools to predict the functional impact of NOTCH pathway mutations: Polyphen-2 (http://genetics.bwh.harvard.edu/pph2/), PROVEAN (http://provean.jcvi.org/seq_submit.php), MutationAssessor (http://mutationassessor.org/r3/), SIFT (http://sift.bii.a-star.edu.sg/www/SIFT_seq_submit2.html), Condel (http://bg.upf.edu/fannsdb/query/condel) and SuSPect (http://www.sbg.bio.ic.ac.uk/~suspect/index.html). Data are summarized in Table S3.

***RNA extraction, cDNA synthesis and semi-quantitative reverse-transcription polymerase chain reaction (qRT-PCR)***

Total RNA of cell lines was extracted with NucleoSpin RNA Kit (Macherey-Nagel, Düren, Germany) according to the manufacturer’s protocol. For RNA extraction of murine tissues, cryopreserved tissues were homogenized with Precellys CKMix Tissue Homogenizing Kit (Bertin-Corp, Rockville, MD, USA), followed by RNA isolation with ExtractMe Total RNA Kit (Blirt, Gdansk, Poland) according to the manufacturer’s protocol. cDNA was synthesized from 0.5 to 1 μg total RNA using RevertAid H Minus First Strand cDNA Synthesis Kit (Thermo Fisher Scientific). Samples of at least three independent experiments were analyzed in duplicates using primaQuant (Steinbrenner Laborsyteme GmbH, Wiesenbach, Germany) on a StepOnePlus real-time PCR instrument (Thermo Fisher Scientific). The human or murine reference gene serine/arginine-rich splicing factor 4 (*SRSF4*) was used as an internal control. Relative mRNA expression values were calculated using the comparative Ct method. Primers were obtained from Thermo Fisher Scientific and are listed in Table S7.

***Gene expression microarray and data analysis***

For gene expression profiling, uninfected Hep3B control cells, Hep3B-HES5wt and Hep3B-HES5mut cells with or without 2 µg/ml doxycycline (Dox) treatment were used. RNA quality was tested by capillary electrophoresis using the Agilent 2100 bioanalyzer (Agilent). Gene expression profiling was performed using arrays of Clariom D Human microarrays (Thermo Fisher Scientific). Biotinylated antisense cDNA was then prepared according to the standard labelling protocol with the GeneChip® WT Plus Reagent Kit and the GeneChip® Hybridization, Wash and Stain Kit (both from Thermo Fisher Scientific). Afterwards, the hybridization was performed in a GeneChip Hybridization oven 640, microarrays were dyed in the GeneChip Fluidics Station 450 and thereafter scanned with a GeneChip Scanner 3000. All of equipment used was from Affymetrix (Affymetrix, High Wycombe, UK). Custom CDF Version 22 with ENTREZ based gene definitions was used to annotate the arrays [14]. The raw fluorescence intensity values were normalized applying quantile normalization and Kernel Surface background correction. OneWay-ANOVA was performed to identify differential expressed genes using a commercial software package SAS JMP10 Genomics, version 6, from SAS (SAS Institute, Cary, NC, USA). Genes significantly different between +Dox and –Dox control of Hep3B-HES5wt inducible cells (N=2132, FDR p-value<0.05) were selected to examine significant enrichment in functionally related categories including KEGG pathways, by means of using DAVID functional annotation tool with default parameters [15, 16].

***Construction of expression vectors***

Codon-optimized HES5-R31G mutant cDNA constructs subcloned into pDONR221 were obtained from GeneArt Synthesis (Thermo Fisher Scientific) and mutagenized into HES5wt, HES5-S34D and HES5-S35D as described above (Table S8). pDONR constructs were then cloned into pDEST26-C-FLAG for transient expression or into the inducible lentiviral vector pTRIPZ-GW via Gateway Technology (Thermo Fisher Scientific) according to the manufacturer’s protocol.

***Lentivirus production, target cell infection and positive selection of inducible cell lines***

Twenty-four hours before transfection, HEK293T packaging cells were seeded in poly-L-lysine coated 10 cm plates and cultured in growth medium to achieve ∼70% confluency. For transfection, 10 μg of the respective pTRIPZ vector, 8 µg psPAX2 and 2.5 µg pMD2.G were co-transfected with 60 µl PEI. Cells were incubated at 37°C with 5% CO_2_ overnight and medium was removed. Viral particle-containing supernatants were harvested at 48 h and 72 h and pooled supernatants were filtered using 0.45 μm Millex-HA filter (Merck Millipore, Burlington, MA, USA). Hep3B and SNU475 cell lines were infected with virus supernatant and 48 h post-infection cells were selected with 1 µg/ml puromycin for at least one week.

***Cell viability assay***

To analyze cell viability, 10,000 Hep3B cells or SNU475 cells were seeded in 12-well plates in triplicates, induced with 2 µg/ml or 0.5 µg/ml doxycycline, respectively, and viability was measured every 24 h for 3 days. Growth medium containing 10% Resazurin (R&D Systems, Minneapolis, MN, USA) was added to the cells, incubated for 1 h at 37°C and measured (544 nm Ex/590 nm Em) with an Omega FLUOstar Microplate Reader (BMG LABTECH, Ortenberg, Germany).

***Colony formation assay***

For colony formation, 1,000 cells were seeded in 6-well plates in triplicates and cultured with or without 2 µg/ml or 0.5 µg/ml doxycycline for 12–15 days. Then, cells were washed with ice-cold PBS and stained with 0.5% crystal violet solution in 25% methanol for 30-60 min at room temperature. All experiments were conducted at least three times.

***Cell migration assay***

Time-lapse microscopy was used to monitor lateral cell migration. For Hep3B, 150,000 cells and for SNU475, 125,000 cells were seeded in 12-well plates and induced with 2 µg/ml (Hep3B) or 0.5 µg/ml (SNU475) doxycycline for 24 h. Cell proliferation was inhibited by treating the cells with 5 µg/ml Mitomycin C (Pharmacy, University Hospital Heidelberg) for 3 h. A cell gap was created using a sterile 10µl-tip (Steinbrenner Laborsysteme GmbH). Subsequently, the cells were washed twice with PBS and cultured with medium containing 20 ng/µl HGF to promote migration and with or without doxycycline. Directional cell migration towards the gap was documented in a time-resolved manner for up to 24 h by the IX81 microscope in the Cell^R imaging station (Olympus, Hamburg, Germany). The gap area was quantified at specific time points by the image software analysis Fiji and relative cell migration (M) was calculated as follows: M=(1-GXh/G0h)experimental/(1-GXh/G0h)control; where G represents the quantification of the gap area.

***ß-galactosidase assay***

Hep3B cells were seeded in 6-well plates and treated with 2 µg/ml doxycycline for 4 days. After washing the cells with PBS the cells were fixed with 0.5% glutaraldehyde/PBS solution for 15 min at room temperature, washed with 1 mM MgCl2 in PBS pH 6.0 and incubated with X-Gal staining solution (1 mg/ml X-Gal, 5 mM potassium hexacyanoferrate(II) trihydrate, 5 mM potassium hexacyanoferrate(III)) for 4-6 h at 37°C. Images were taken at the Nikon Imaging Center Heidelberg with a Nikon NiE widefield microscope, Nikon Plan Apo λ 10x NA 0.45 objective and captured using Nikon DS-Ri2 color camera (Nikon). For quantification, four images per treatment group were acquired with 4908 x 3264 pixel frames. The area of ß-galactosidase-positive (senescent) cells and the area of unstained cells were segmented and determined using ilastik software [17]. Further quantification of the ratio of senescence and normal cells area was done using Fiji macro and then statistically analyzed.

***Chromatin immunoprecipitation (ChIP)***

Hep3B cells were grown on 15 cm plates with or without doxycycline induction (2 µg/ml) for 2 days. For ChIP experiments, the SimpleChIP® Enzymatic Chromatin IP Kit with magnetic beads (Cell Signaling, Frankfurt, Germany) was conducted according to the manufacturer’s protocol. Binding to genomic loci was analyzed with qRT-PCR and Ct values of the ChIP samples were normalized to the respective input. As negative control, primers flanking a region of genomic DNA between the *GAPDH* gene and the chromosome condensation-related SMC-associated protein (*CNAP1*) gene were used (Chip‑it kit, Active Motif, Carlsbad, CA, USA) as well as primers in the 3-prime untranslated region of the analyzed gene promoter. For primers see Table S9.

***ENCODE MYC ChIP-seq data analysis***

MYC ChIPseq peakfile data of human HepG2 cells was downloaded from ENCODE database (https://www.encodeproject.org/experiments/ENCSR000DLR/). Location of the peaks was annotated with ChipSeeker on hg19 annotation [18].

***Western Blot and cell fractionation***

Total protein was extracted from cell lines with cell lysis buffer (Cell Signaling) supplemented with PhosStop and protease inhibitor Complete Mini EDTA-free (Roche Diagnostics, Mannheim, Germany). For cytoplasmic and nuclear cell fractionation, the NE-PER Nuclear and Cytoplasmic Extraction Kit (Thermo Fisher Scientific) was used according to the manufacturer’s protocol. The protein concentration of each sample was determined by Bradford assay (Sigma-Aldrich). Twenty-five μg of protein lysates were separated on 8% or 15% Bis/Tris-polyacrylamide gels and then transferred to a nitrocellulose membrane (Amersham Biosciences, Buckinghamshire, UK). Membranes were blocked with 5% milk or 5% bovine serum albumin in TBST and immunoblotted with the indicated antibodies overnight at 4°C (Table S10). Proteins were detected with IRDye secondary antibodies using an Odyssey Sa Infrared Imaging System (LI-COR Biosciences, Bad Homburg, Germany). Protein abundance was quantified using Image Studio v3.1.4 (LI-COR Biosciences).

***Co-immunoprecipitation (co-IP)***

To identify protein-protein interactions co-immunoprecipitation (co-IP) was performed. HEK293T cells were transfected with Lipofectamine 2000 transfection reagent (Life Technologies) and after 48 h harvested in non-denaturing lysis buffer (50 mM Tris-HCl pH 7.4, 1% NP-40, 0.25% sodium deoxycholate, 150 mM NaCl, 1 mM EDTA) supplemented with 1 mM PMSF, 1 mM DTT, PhosStop and protease inhibitor Complete Mini EDTA-free. Anti-FLAG M2 mouse antibody (Sigma-Aldrich) or negative control mouse IgG1 (Dako) with Protein G Dynabeads (Thermo Fisher Scientific) were incubated for 1.5 h at 4°C while rotating. After gentle washing with PBST 1 mg of protein lysate was added and incubated for 2 h at 4°C while rotating. After three washing steps immunoprecipitated proteins were eluted by incubating beads in 1x sample buffer (62.5 mM Tris HCl pH 6.8, 2% SDS, 10% glycerol, 0.01% bromophenol blue, 25 mM DTT) at room temperature for 20 min while shaking. Supernatants were boiled for 8 min at 95°C and separated by SDS-PAGE. Detection was carried out with anti-AKT (Cell Signaling), anti-pAKT-Ser473 (Cell Signaling), anti-HES1 (Cell Signaling) or anti-HES5 (Atlas Antibodies, Bromma, Sweden).

***Proximity Ligation Assay (PLA) and immunofluorescence***

PLA and immunofluorescence were performed as previously reported [19]. Briefly, glass cover slips were coated with poly-L-lysine for 30 min at 37°C before seeding of cells. Forty-eight hours post-induction, cells were fixed in 4% PFA for 10 min and permeabilized with 0.2% Triton X-100/PBS for 7 min. PLA was performed with the DUOLINK assay (Sigma-Aldrich) according to the manufacturer’s protocol. Cells were incubated with anti-HES5 and anti-AKT antibodies for 1.5 h at room temperature. To detect the protein-protein interactions the Duolink In Situ Detection Kit Orange (Sigma-Aldrich) was used. For staining F-actin, cells were incubated with 50 µl CytoPainter Phalloidin-iFluor 488 Reagent (ab176753, Abnova, Taipei, Taiwan) for 30 min at room temperature according to the manufacturer’s protocol. Cover slips were air dried and mounted with Duolink *In Situ* Mounting Medium with DAPI (Sigma-Aldrich). Cells were examined with a Nikon C2+ confocal microscope (Nikon, Duesseldorf, Germany) at the Nikon Imaging Center Heidelberg. For quantification of protein-protein interaction, 8 images with at least 10 cells per image were taken with 2048 x 2048 pixel frames. The dots per cell indicating protein-protein interaction were manually quantified using Fiji [20].

For immunofluorescence, cells were fixed and permeabilized as mentioned above. The slides were blocked with 1% BSA/PBS for 30 min, followed by the primary anti-HES5 antibody incubation in a wet chamber at room temperature for 1.5 h. The secondary antibody incubation was performed using the Cy3 goat anti-rabbit antibody (Jackson ImmunoResearch Laboratories, Ely, UK) at room temperature for 1 h. Cells were dehydrated with 100% ethanol and mounted with DAPI fluoromount-G (Southern Biotech, Birmingham, USA). Images were taken with a Nikon C2+ confocal microscope (Nikon) and processed with Fiji software. Fluorescence intensity profiles were drawn with the RGB Profile of the Fiji plugin Colour functions with the same parameters for all analyzed images.

***Immunohistochemistry (IHC)***

Immunohistochemical analyses were performed on 3 μm thick sections of murine FFPE liver blocks. For Ki67 (ab15580, rabbit, 1:4,000, abcam, Cambridge, MA) and for HNF4A (ab201460, rabbit, 1:4,000, abcam), slides were pretreated by boiling for 10 min with pH 6.0 Buffer (S2031, Agilent) or pH 9.0 Buffer (S2367, Agilent), respectively. An immunoperoxidase method was used to visualize bound antibodies with DAB (K5005, Dako REAL Detection System Peroxidase/AEC, Rabbit/Mouse, Agilent) as chromogen. PanCK (Z0622, rabbit, 1:400, Agilent) was stained after pretreatment for 30 min pH 6.0 Buffer (S1699, Agilent) in a microwave steam cooker. Stained slides were scanned using the NanoZoomer slide scanner (Hamamatsu Photonics, Hamamatsu, Japan) and analyzed by QuPath digital software (<https://qupath.github.io/>).

***Hydrodynamic tail vein injection and animal studies***

The experimental setup and group size was approved by the German Regional Council of Baden‐Wuerttemberg. Exclusion and termination criteria were defined by criteria of the animal welfare officer of the University Hospital Heidelberg. Hydrodynamic tail vein injection was performed as previously described [21]. Murine Hes5 cDNA (TetO-FUW-Hes5) was a gift from Rudolf Jaenisch (Addgene, Watertown, MA, USA, plasmid #61536) and was cloned into pT3-EF1ɑ-IRES-GFP. In addition, murine Hes5-R31G (Hes5mut) cDNA was generated by site-directed mutagenesis using AccuPOL DNA polymerase (VWR, Bruchsal, Germany) and mutagenesis primers, followed by DpnI digest (Thermo Fisher Scientific). To induce liver tumorigenesis, 10 μg pT3-EF1α-MYC or pT3-EF1α-HA-myrAKT were combined with 10 μg pT3-EF1α-Hes5wt-IRES-GFP or pT3-EF1α-Hes5mut-IRES-GFP and together with a 5:1 ratio of pT3 plasmids over Sleeping Beauty transposase (pCMV-SB10) in 2 ml PBS and injected into the tail veins of 8-9 weeks old male and female FVB/N mice within 7-10 seconds (Charles River Laboratories, Sulzfeld, Germany). At the indicated time points, mouse livers were isolated and stored in liquid nitrogen or fixed in buffered formalin for further analysis.

**Supplemental Figures**

**
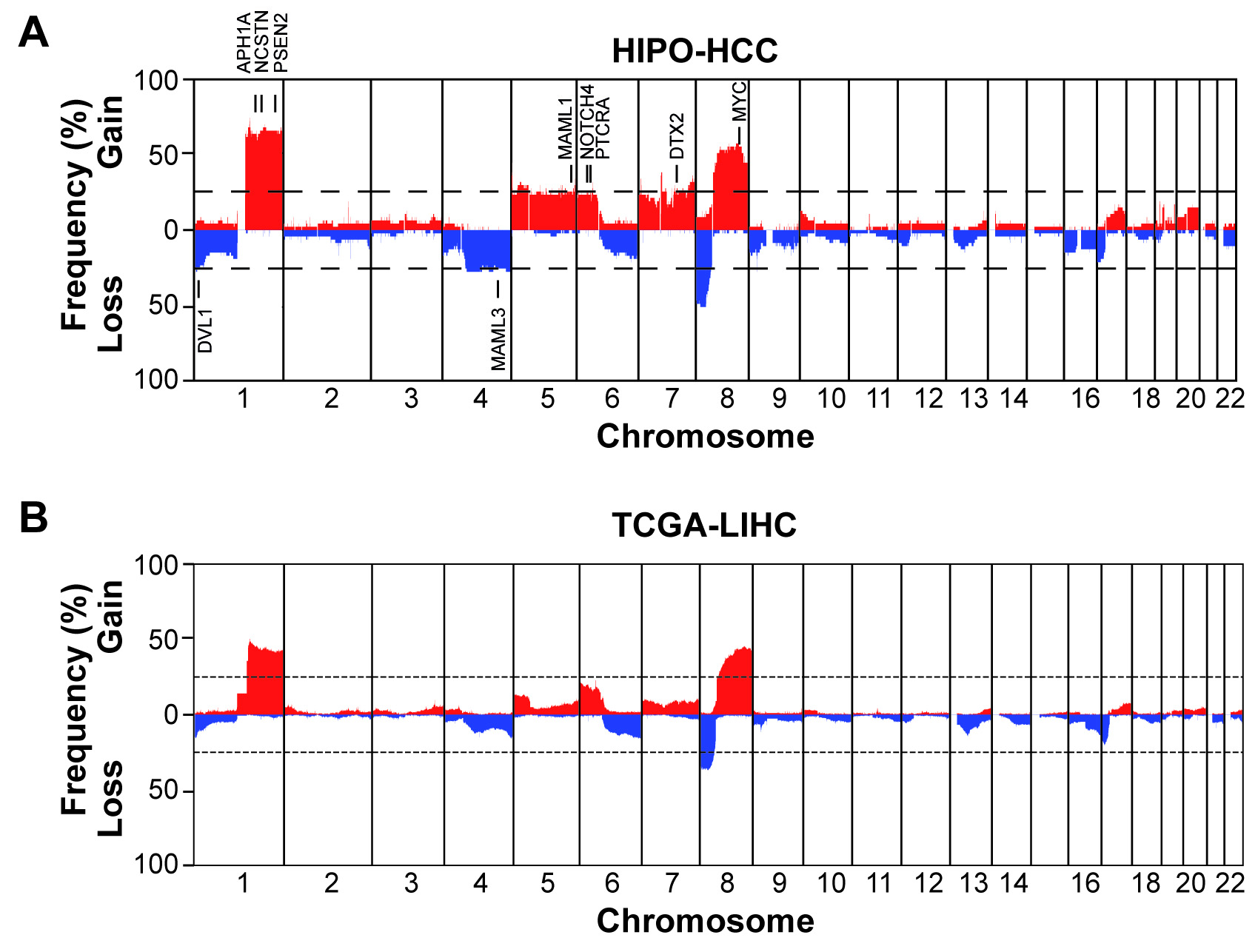
**

**Figure S1: Copy number alterations of NOTCH pathway genes and MYC in HCC. (A)** Frequency of copy number alterations observed in the whole exome sequencing data of the HIPO cohort (N=47). Chromosome boundaries are indicated by vertical solid lines and horizontal dashed lines imply a frequency of 20%. The genomic location of affected NOTCH pathway genes and of MYC is depicted. **(B)** Copy number alterations observed in the TCGA-LIHC cohort (N=384).

**
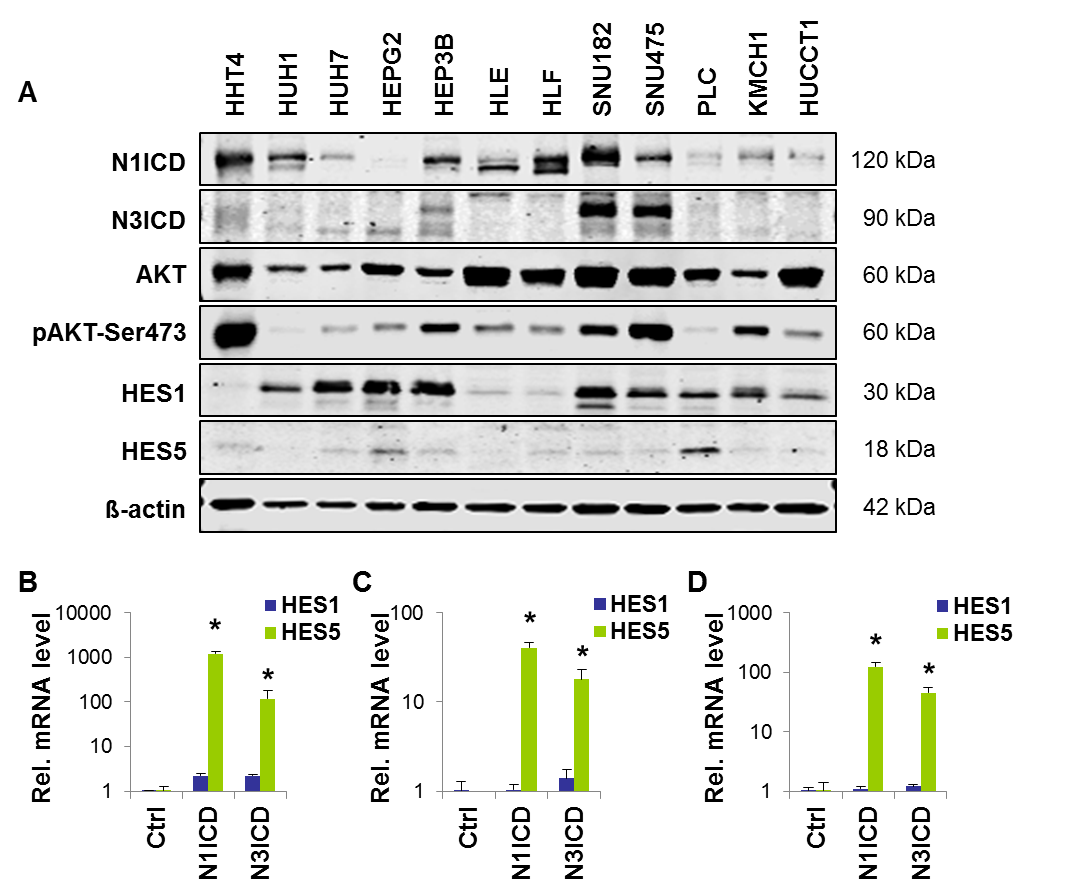
**

**Figure S2: NOTCH signaling induces HES1 and HES5 mRNA in liver cancer cell lines. (A)** Liver cancer cell lines express varying levels of NOTCH1, NOTCH3, AKT, phospho-AKT (Ser473), HES1 and HES5 protein levels as illustrated by Western blot. Noteworthy, the cell lines predominantly exhibited cleaved active forms N1ICD and/or N3ICD. β-actin served as loading control. **(B)** Hep3B, **(C)** HuH1 and **(D)** KMCH1 cells were transiently transfected with pDEST-control (Ctrl), pDEST-N1ICD or pDEST-N3ICD followed by qRT-PCR of HES1 and HES5 mRNA after 48 h.

**
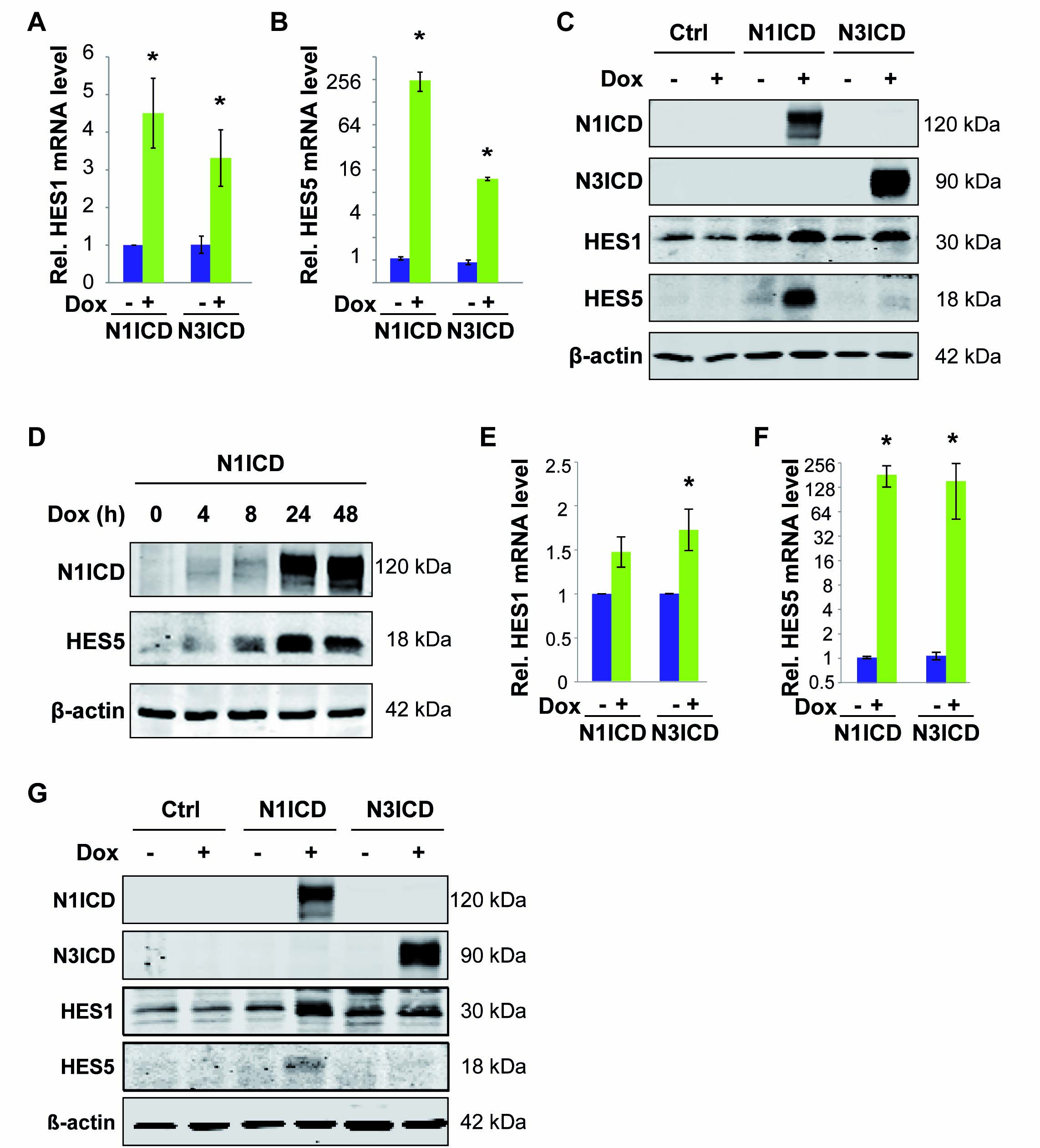
**

**Figure S3: NOTCH signaling induces HES1 and HES5 expression in HCC cells.** **(A and B)** Quantitative RT-PCR of Hep3B cells upon induction of N1ICD or N3ICD protein by 2 µg/ml Doxycycline (Dox) reveals upregulation of HES1 **(A)** and HES5 **(B)** mRNA expression, with N=3 or N=2, respectively. **(C)** Western blot showing HES1 and HES5 protein levels upon induction of N1ICD or N3ICD in Hep3B cells by 2 µg/ml Dox for 48 h. **(D)** Time course experiment illustrating HES5 expression after N1ICD induction in Hep3B cells for indicated time points. **(E and F)** Induction of N1ICD or N3ICD protein by 0.5 µg/ml Dox leads to upregulation of HES1 and HES5 mRNA expression in SNU475 cells (N=3), respectively. **(G)** Western blot showing HES1 and HES5 protein levels upon N1ICD or N3ICD induction in SNU475 cells treated with 0.5 µg/ml Dox for 48 h. For all Western blots one representative experiment out of three with similar outcome is shown. β-actin served as loading control.

**
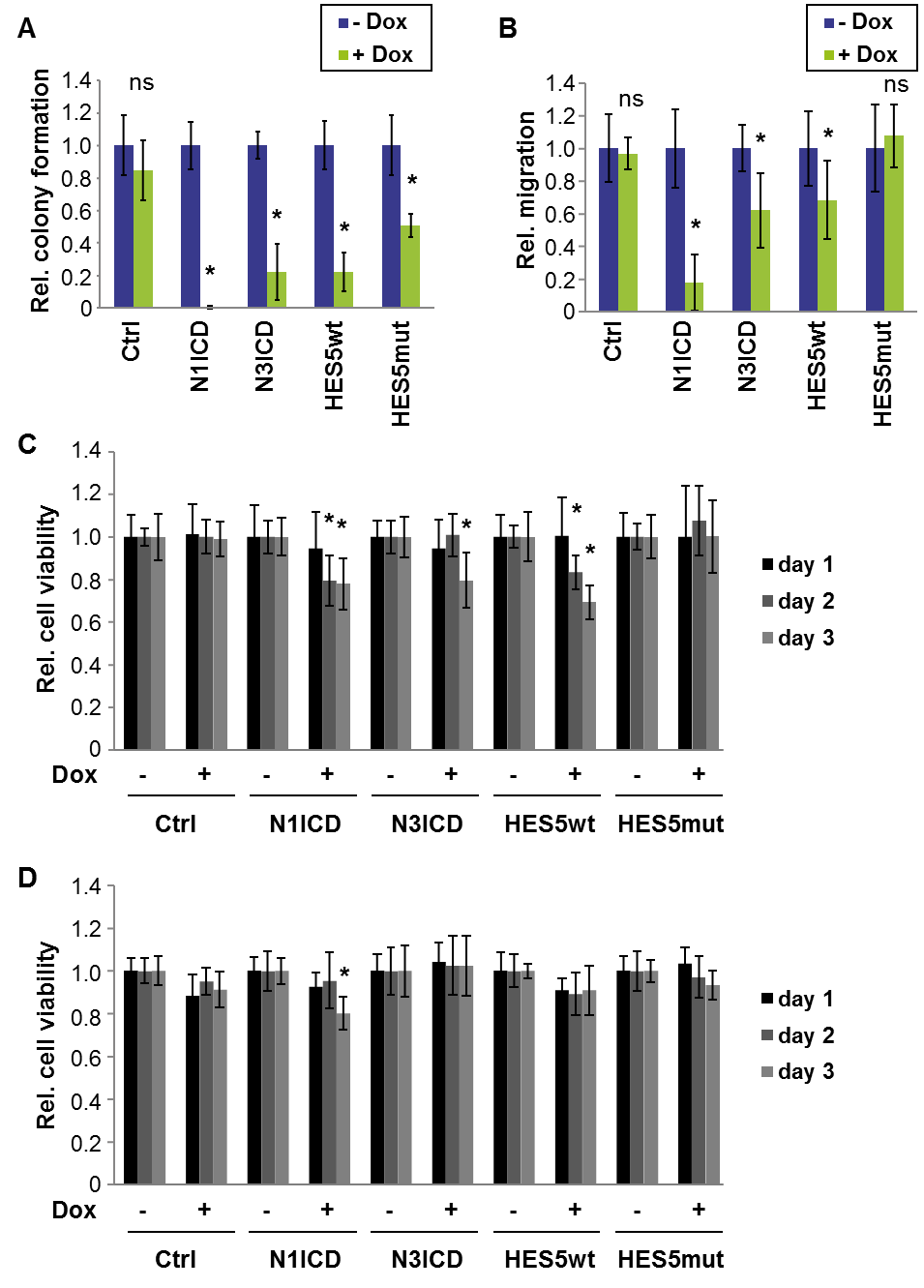
**

**Figure S4: HES5wt but not HES5mut reduces colony formation and cell migration of HCC cells. (A)** Quantification of relative colony formation of SNU475 treated with 0.5 µg/ml Dox or left untreated (N=2). **(B)** Relative migration of SNU475 cells treated with 0.5 µg/ml Dox or left untreated (N=4). **(C)** Cell viability of Hep3B (N=5) and **(D)** SNU475 (N=4) was measured for 3 days with or without 2 µg/ml or 0.5 µg/ml Dox, respectively. Relative mean values with SD are shown; * Mann-Whitney U test p<0.05; ns=not significant.

**
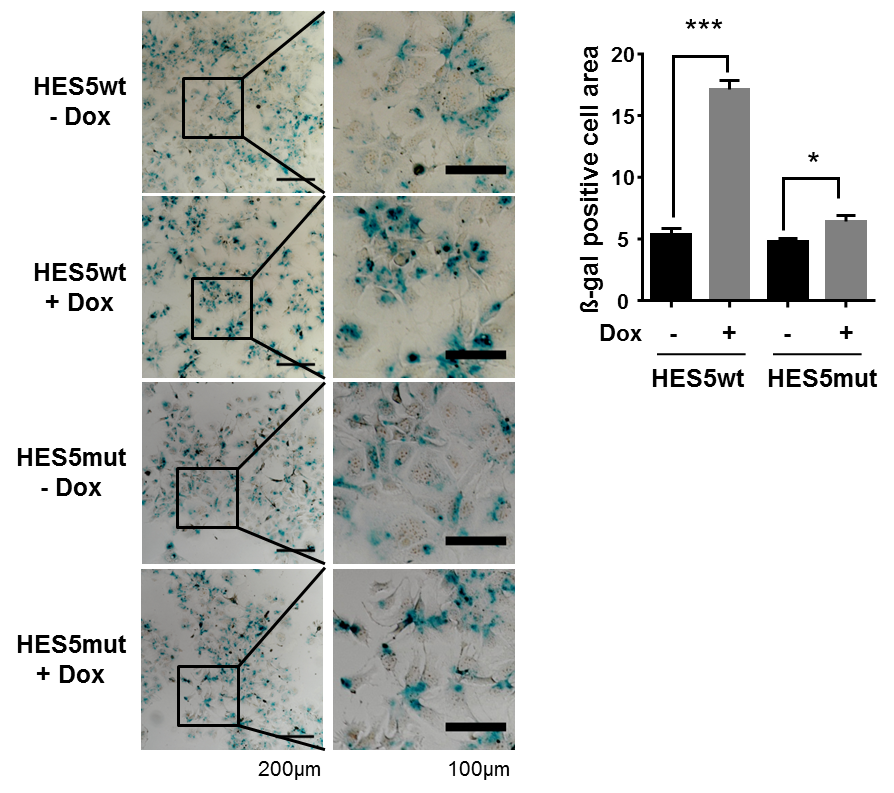
**

**Figure S5: HES5wt but not HES5mut induces cellular senescence.** Representative images of β-galactosidase staining of Hep3B-HES5wt or Hep3B-HES5mut cells with or without Dox treatment (2 µg/ml) for 4 days. The histogram shows the percentage of cellular area with β-galactosidase positive staining (blue); images of one out of 4 representative experiments. *** Mann-Whitney U test p<0.001, * Mann-Whitney U test p<0.05.


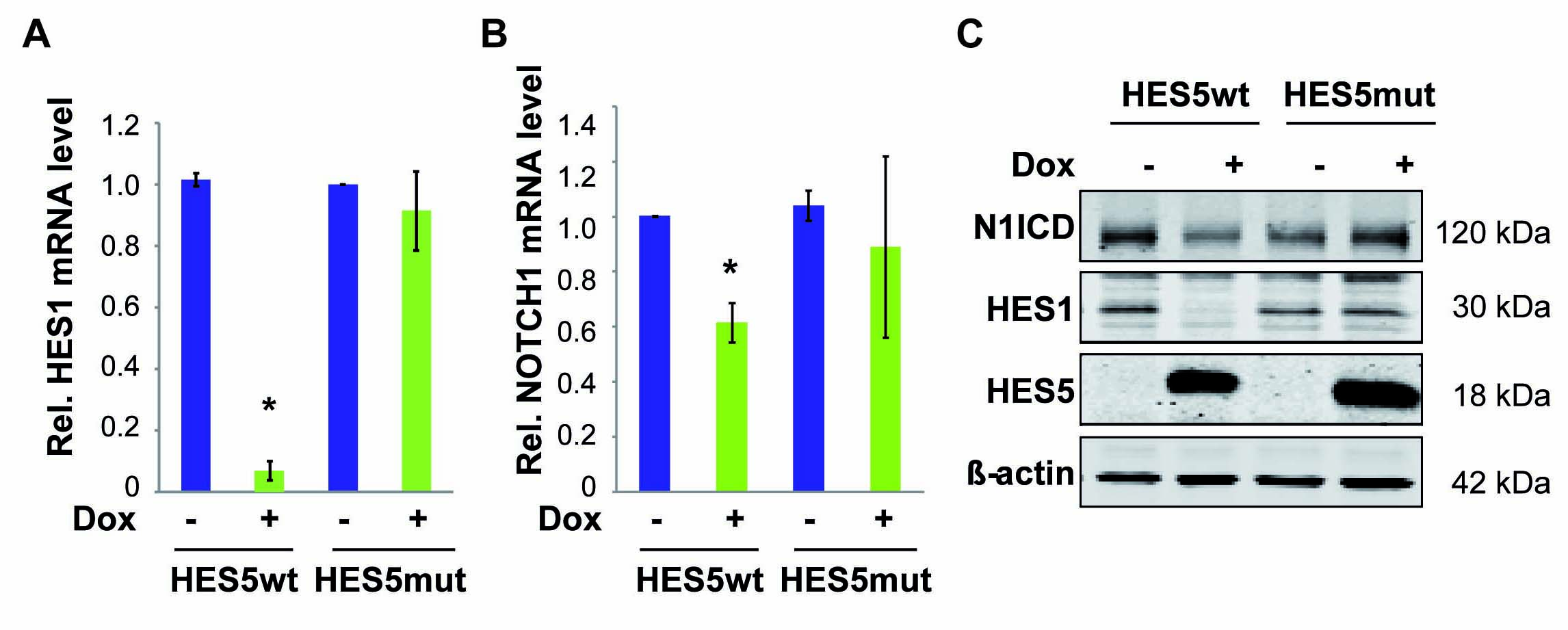


**Figure S6: HES5wt but not HES5mut alters gene expression in SNU475 cells. (A and B)** Quantitative RT-PCR of SNU475 cells upon HES5wt or HES5mut induction by 0.5 µg/ml Dox showing repression of HES1 and NOTCH1 (N=3). * Mann-Whitney U test p<0.05. **(C)** Western blot detecting HES1, HES5 and N1ICD protein levels in SNU475 cells upon HES5wt or HES5mut expression by 0.5 µg/ml Dox treatment. One representative experiment out of three with similar outcome is shown. β-actin served as loading control.


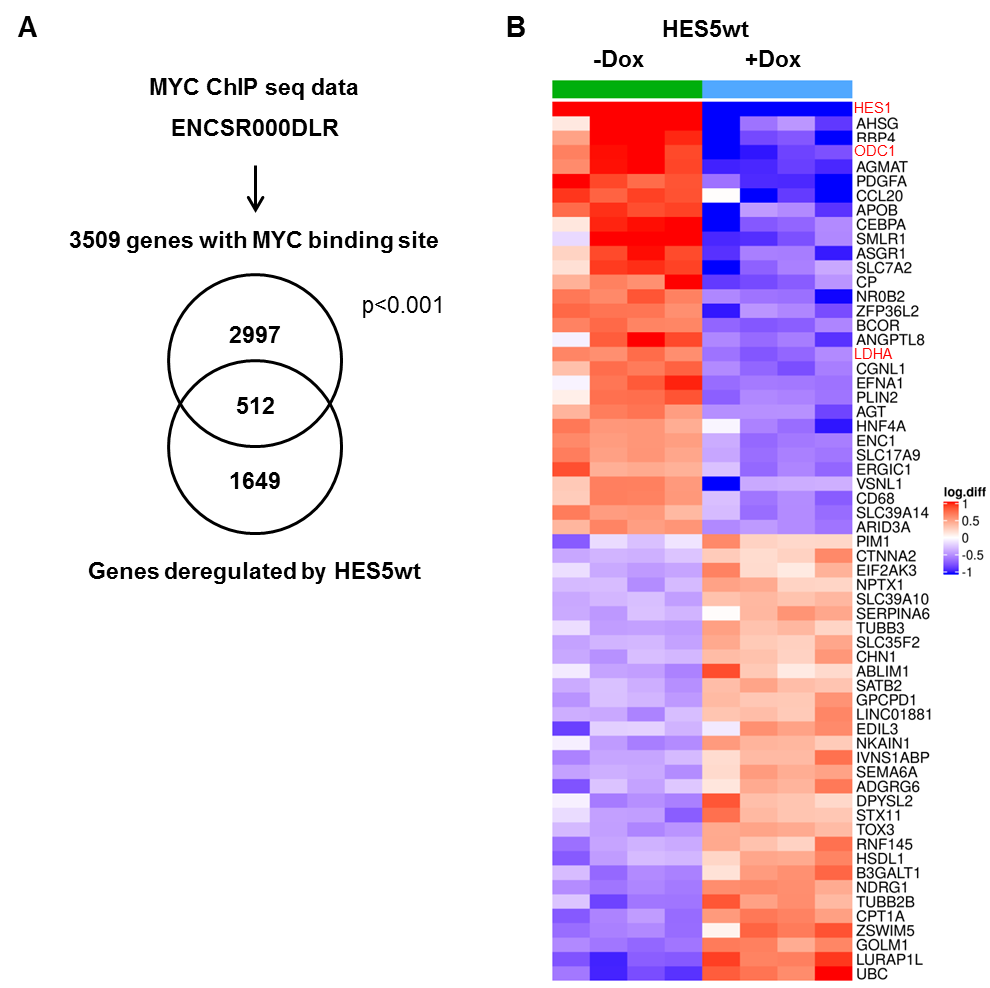


**Figure S7: MYC target genes are enriched among HES5 target genes.** **(A)** Schematic diagram of data analysis. Using ENCODE MYC ChIP seq data ENCSR000DLR of HepG2 liver cancer cells, we identified peaks in 3509 genes. Of these, 512 genes were significantly deregulated by HES5wt (adj. p<0.05). **(B)** Heatmap of the top up and down regulated HES5 target genes with MYC binding site. HES1, ODC1 and LDHA are highlighted in red.


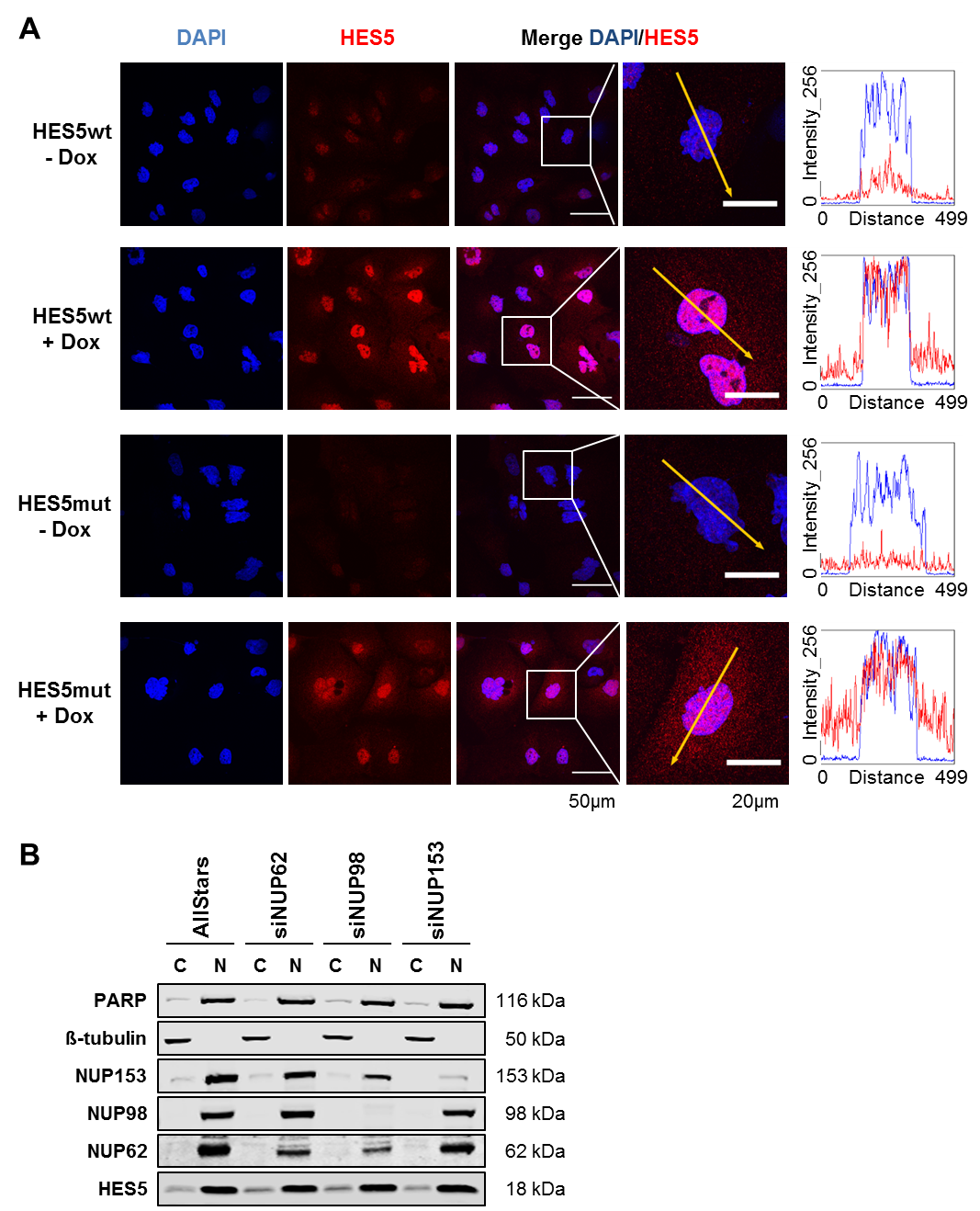


**Figure S8: HES5mut protein is more abundant in the cytoplasm than HES5wt. (A)** Representative images of HES5 immunofluorescence in HES5wt or HES5mut expressing SNU475 cells with or without 0.5 µg/ml Dox treatment as indicated. The yellow arrow (50 µm long) in the magnified image represents intensity profiles of HES5 (red signal) and DAPI (blue signal).**(B)** Western blot displaying nuclear pore protein levels and HES5 localization in cytoplasm (C) and nucleus (N) upon siRNA-mediated knockdown of NUP62, NUP98 and NUP153 or transfection of AllStars control. PARP and ß-tubulin served as markers for nuclear or cytoplasmic compartments, respectively.

**
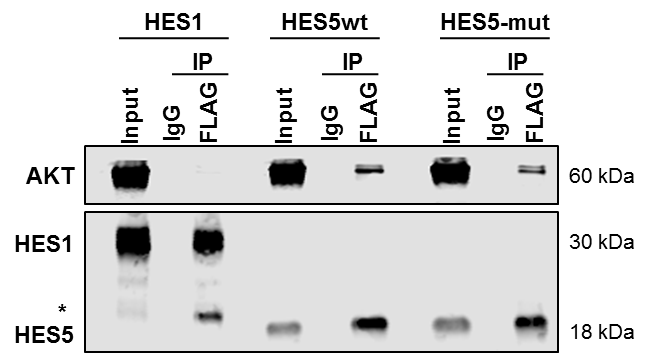
**

**Figure S9: HES5wt and HES5mut but not HES1 directly bind AKT.** Co-immunoprecipitation experiments of HEK293T cells transiently co-transfected with HES1-FLAG (left), HES5wt-FLAG (middle) or HES5mut-FLAG (right) together with AKT. After anti-FLAG immunoprecipitation, samples were separated by SDS–PAGE and subjected to Western blot analysis with anti-AKT, anti-HES1 or anti-HES5-reactive antibodies. One representative experiment out of three with similar outcome is shown. * unspecific band.


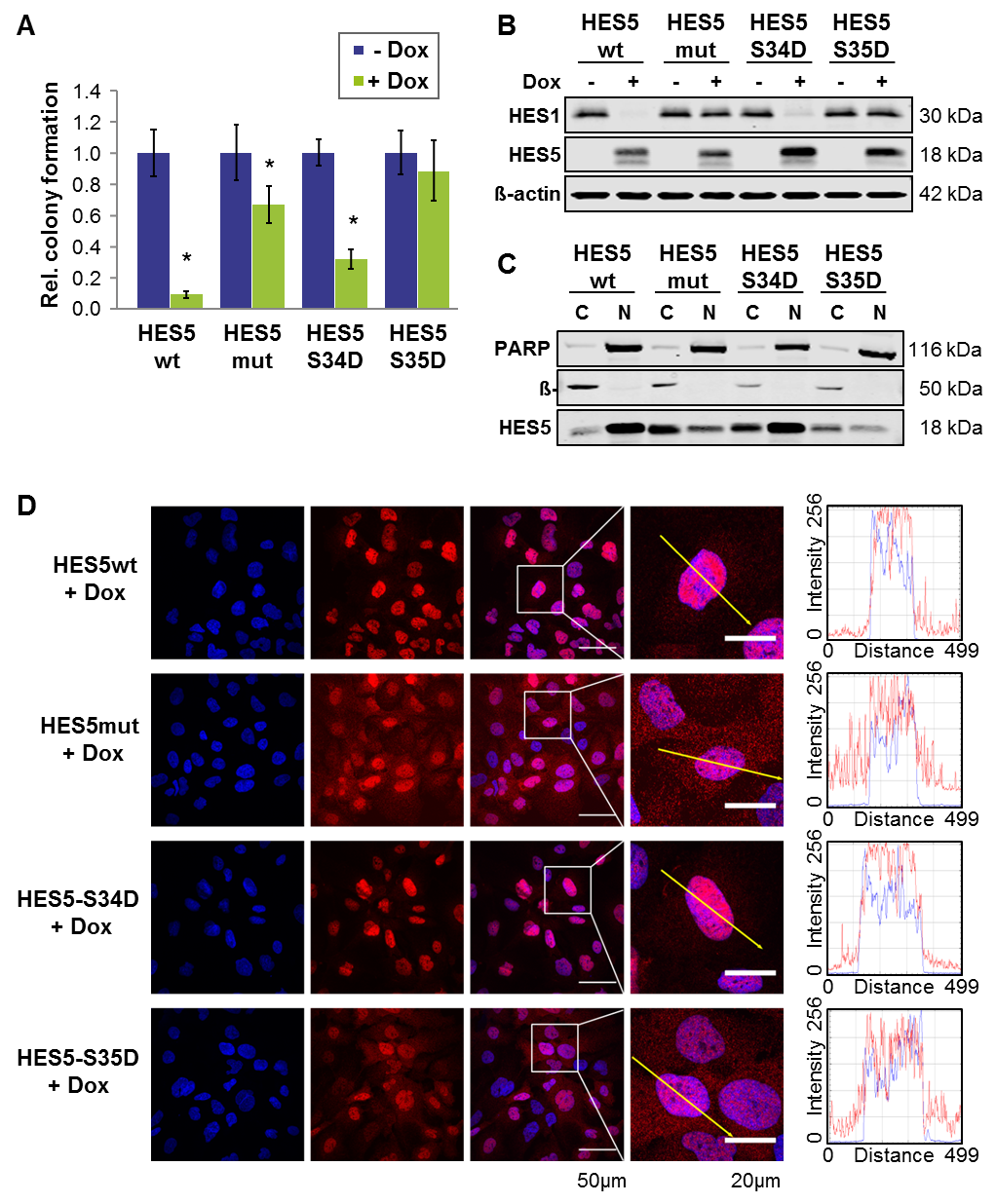


**Figure S10: Function and localization of HES5wt, HES5mut and the phosphomimicing HES5 variants HES5-S34D and HES5-S35D. (A)** Relative colony formation of the indicated Hep3B cells treated with 2 µg/ml Dox or left untreated (N=3). **(B)** Protein levels of HES1 after induction of HES5 with 2 µg/ml Dox treatment in Hep3B-HES5wt, Hep3B-HES5mut (R31G), Hep3B-HES5-S34D and Hep3B-HES5-S35D shown by Western blot analysis. **(C)** Cell fractionation for HES5 localization in the indicated Hep3B cell lines. PARP and ß-tubulin served as markers for nuclear or cytoplasmic compartments, respectively. For all Western blots one representative experiment out of three with similar outcome is shown. **(D)** Representative images of indicated Hep3B cell lines with 2 µg/ml Dox treatment as indicated. The yellow arrow (50 µm long) in the magnified image represents intensity profiles of HES5 (red signal) and DAPI (blue signal). * Mann-Whitney U test p<0.05.

**
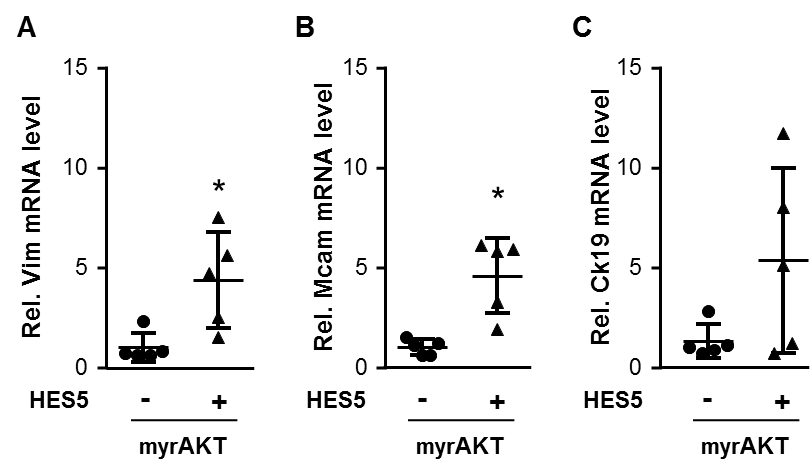
**

**Figure S11: Induction of EMT and differentiation markers in murine AKT-driven tumors.** Quantitative RT-PCR of Vim **(A)**, Mcam **(B)** and Ck19 **(C)** mRNA levels from isolated mouse liver tissues transduced with myrAKT (N=5) or myrAKT/HES5wt (N=5). * Mann-Whitney U test p<0.05.


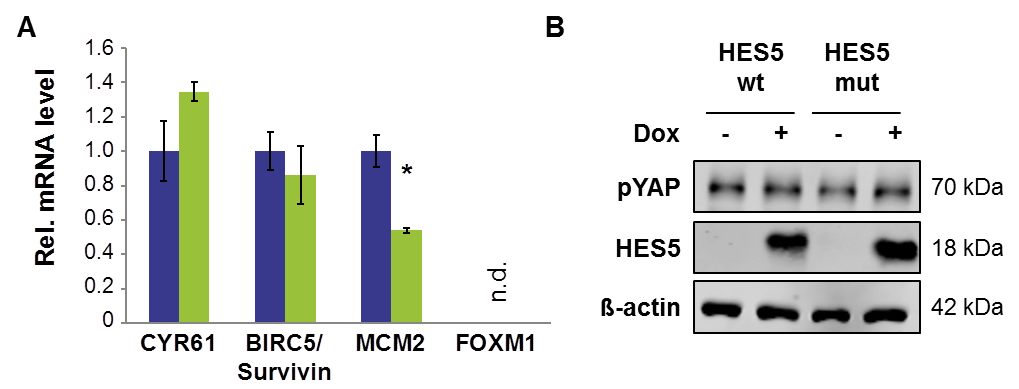


**Figure S12: YAP signaling upon HES5 induction. (A)** Quantitative RT-PCR of Hep3B-HES5wt cells treated with 2 µg/ml Dox or left untreated (N=4). FOXM1 was not detectable in Hep3B cells. n.d. not detectable; * Mann-Whitney U test p<0.05. **(B)** Western blot of phospho-YAP (Ser127), HES5 and ß-actin of Hep3B-HES5wt and HES5mut cells with or without treatment as indicated. One representative Western blot experiment out of three with similar outcome is shown.

**Supplemental Tables**

**Table S1: Patient characteristics of HCC cohort (N=54) used for whole exome sequencing.** For 47 patients, CNA was performed. NOTCH pathway component, TP53 and CTNNB1 mutations as well as MYC amplification are indicated.

| **ID** | **Sex** | **G** | **pT** | **UICC** | **R** | **Cirrhosis** | **Etiology** | **CNA*** | **NOTCH pathway** | **TP53** | **CTNNB1** | **MYC**  **amplification** |
| --- | --- | --- | --- | --- | --- | --- | --- | --- | --- | --- | --- | --- |
| HD005 | m | G2 | T1 | 1 | R0 | No | HBV, HCV | Yes | none | wt | wt | Yes |
| HD006 | m | G2 | T1 | 1 | R0 | No | non-viral | Yes | HES5p.R31G; DTX1p.P138L | wt | p.K335I | No |
| HD010 | m | G1 | T2 | 2 | R0 | Yes | C2 | Yes | none | wt | wt | Yes |
| HD011 | m | G2 | T1 | 1 | R0 | Yes | C2 | Yes | NUMBp.P267A | wt | wt | Yes |
| HD012 | f | G2 | T3 | 3 | R0 | No | non-viral | Yes | none | wt | p.K335I | No |
| HD013 | f | G3 | T3 | 3 | R0 | Yes | HBV | Yes | none | wt | wt | Yes |
| HD014 | m | G2 | T4 | 4 | R0 | No | non-viral | Yes | none | p.P27S | wt | No |
| HD015 | m | G2 | T3 | 3 | R0 | Yes | HBV | Yes | none | wt | wt | No |
| HD018 | m | G2 | T2 | 2 | R0 | Yes | non-viral | Yes | none | wt | wt | No |
| HD019 | m | G3 | T3 | 3 | R0 | Yes | C2 | Yes | none | wt | wt | Yes |
| HD021 | m | G4 | T3 | 3 | R0 | Yes | HBV | Yes | none | wt | wt | No |
| HD022 | m | G2 | T2 | 2 | R0 | No | HFE | Yes | GXYLT2p.H367P | wt | p.S45A | Yes |
| HD024 | f | G3 | T2 | 2 | R0 | No | non-viral | Yes | none | wt | wt | No |
| HD026 | m | G1 | T2 | 2 | R0 | Yes | HCV | Yes | none | p.R141C | wt | No |
| HD027 | m | G2 | T1 | 1 | R0 | Yes | HCV | Yes | none | wt | p.H36P | No |
| HD028 | m | G2 | T2 | 2 | R0 | Yes | HCV | No | none | wt | wt | No |
| HD029 | f | G2 | T1 | 1 | R0 | No | HCV | Yes | none | p.E154G | wt | No |
| HD030 | m | G2 | T2 | 2 | R0 | Yes | HCV | Yes | NOTCH3p.Q731X; DLL1p.L264fs | wt | wt | Yes |
| HD032 | m | G2 | T2 | 2 | R0 | Yes | HCV | Yes | none | wt | wt | Yes |
| HD033 | f | G2 | T1 | 1 | R0 | Yes | HCV | Yes | DTX4p.V327F; NCSTNp.L376P;  NOTCH4p.N1926K | wt | wt | Yes |
| HD034 | m | G2 | T1 | 1 | R0 | No | non-viral | Yes | none | wt | p.S45F | No |
| HD037 | m | G2 | T3 | 3 | R1 | No | C2 | Yes | none | wt | p.N387K | Yes |
| HD053 | f | G2 | T3 | 4 | R0 | Yes | C2 | Yes | none | wt | wt | Yes |
| HD055 | m | G3 | T1 | 1 | R0 | No | C2 | Yes | none | Splice | wt | No |
| HD056 | m | G2 | T3 | 3 | NA | No | HBV | No | none | wt | wt | No |
| HD057 | m | G2 | T2 | 2 | R0 | Yes | α1-Antitrypsin | Yes | DMXL2p.T2810A | wt | p.D32G | No |
| HD058 | m | G2 | T2 | 2 | R0 | Yes | C2 | Yes | none | p.R110L | wt | Yes |
| HD059 | m | G2 | T1 | 1 | R0 | Yes | HBV, HCV | Yes | NOTCH3p.C873S; DVL2p.S170C | wt | p.G34V | Yes |
| HD060 | m | G2 | T2 | 2 | R0 | Yes | C2 | No | none | wt | wt | No |
| HD061 | m | G2 | T1 | 1 | R0 | Yes | HBV | Yes | none | p.H61R | wt | Yes |
| HD062 | m | G3 | T3 | 3 | R0 | No | non-viral | Yes | none | p.W14X | wt | Yes |
| HD065 | m | G2 | T2 | 2 | R0 | No | non-viral | No | none | wt | wt | No |
| HD101 | f | G1 | T3 | 3 | NA | No | non-viral | Yes | none | wt | wt | No |
| HD102 | m | G2 | T2 | 2 | NA | Yes | C2 | Yes | DTX3p.P24T | p.R26H | p.N387K | No |
| HD103 | m | G3 | T2 | 2 | NA | Yes | HBV | Yes | none | p.E153K | wt | No |
| HD104 | m | G2 | T3 | 3 | NA | Yes | non-viral | Yes | none | wt | p.S37C | No |
| HD105 | m | G3 | T3 | 3 | NA | No | HBV | Yes | NOTCH3p.E787K | wt | p.S33C | Yes |
| HD106 | m | G3 | T3 | 3 | NA | No | HBV | No | none | wt | wt | No |
| HD107 | m | G3 | T3 | 3 | NA | Yes | HBV | Yes | none | Splice | p.H36P | Yes |
| HD108 | m | G2 | T2 | 2 | NA | Yes | HCV | Yes | LFNGp.I20N | wt | wt | Yes |
| HD204 | f | G2 | T1 | 1 | R0 | No | non-viral | Yes | none | wt | wt | No |
| HD208 | m | G2 | T2 | 2 | NA | Yes | HBV | No | DMXL2p.I2989V | p.V11M | wt | No |
| HD209 | f | G3 | T2 | 2 | R0 | NA | NA | Yes | none | wt | wt | No |
| HD221 | m | NA | NA | NA | NA | NA | NA | Yes | none | wt | wt | No |
| HD227 | m | G2 | T1 | 1 | R0 | NA | NASH | Yes | none | wt | p.S33C | Yes |
| HD229 | f | NA | NA | NA | NA | NA | NA | Yes | DMXL2p.V586A | wt | wt | Yes |
| HD232 | m | G2 | T2 | 2 | R0 | NA | NASH | Yes | NOTCH1p.V1721L; NOTCH1p.A305S | p.C110F | p.K335T; p.W383C | No |
| HD234 | m | G2 | T4 | 4 | R0 | No | HCV | Yes | none | wt | p.G34V | No |
| HD236 | m | G3 | T1 | 1 | R0 | Yes | HCV | Yes | none | wt | p.S45F | Yes |
| HD301 | m | G2 | T2 | 2 | R0 | Yes | non-viral | No | none | wt | wt | No |
| HD302 | m | G2 | T1 | 1 | R0 | Yes | HCV | Yes | none | wt | wt | Yes |
| HD303 | m | G2 | T2 | 2 | R0 | Yes | C2 | Yes | none | wt | wt | Yes |
| HD304 | m | G2 | T3 | 3 | R1 | Yes | C2 | Yes | none | wt | wt | Yes |
| HD306 | f | G1 | T1 | 1 | R0 | Yes | HBV | Yes | none | wt | wt | No |

*CNA: copy number alteration analysis

**Table S2: NOTCH pathway genes (KEGG PATHWAY hsa04330) included in the analysis of genomic alterations.**

| **Gene name** | **ID** | **KEGG ORTHOLOGY** |
| --- | --- | --- |
| ADAM17 | hsa:6868 | K06059 |
| APH1A | hsa:51107 | K06172 |
| APH1B | hsa:83464 | K06172 |
| CIR1 | hsa:9541 | K06066 |
| CREBBP | hsa:1387 | K04498 |
| CTBP1 | hsa:1487 | K04496 |
| CTBP2 | hsa:1488 | K04496 |
| DLL1 | hsa:28514 | K06051 |
| DLL3 | hsa:10683 | K06051 |
| DLL4 | hsa:54567 | K06051 |
| DMXL2 | manually curated |  |
| DTX1 | hsa:182 | K06052 |
| DTX2 | hsa:113878 | K06058 |
| DTX3 | hsa:196403 | K06058 |
| DTX3L | hsa:151636 | K06058 |
| DTX4 | hsa:23220 | K06058 |
| DVL1 | hsa:1855 | K02353 |
| DVL2 | hsa:1856 | K02353 |
| DVL3 | hsa:1857 | K02353 |
| EP300 | hsa:2033 | K04498 |
| GXYLT1 | manually curated |  |
| GXYLT2 | manually curated |  |
| HDAC1 | hsa:3065 | K06067 |
| HDAC2 | hsa:3066 | K06067 |
| HES1 | hsa:3280 | K06054 |
| HES5 | hsa:388585 | K06055 |
| JAG1 | hsa:1840 | K06058 |
| JAG2 | hsa:3714 | K21635 |
| KAT2A | hsa:2648 | K06062 |
| KAT2B | hsa:8850 | K06062 |
| LFNG | hsa:3955 | K05948 |
| MAML1 | hsa:9794 | K06061 |
| MAML2 | hsa:84441 | K06061 |
| MAML3 | hsa:55534 | K06061 |
| MFNG | hsa:4242 | K05948 |
| NCOR2 | hsa:9612 | K06065 |
| NCSTN | hsa:23385 | K06171 |
| NOTCH1 | hsa:4851 | K02599 |
| NOTCH2 | hsa:4853 | K20994 |
| NOTCH3 | hsa:4854 | K20995 |
| NOTCH4 | hsa:4855 | K20996 |
| NUMB | hsa:8650 | K06057 |
| NUMBL | hsa:9253 | K06057 |
| PSEN1 | hsa:5663 | K04505 |
| PSEN2 | hsa:5664 | K04522 |
| PSENEN | hsa:55851 | K06170 |
| PTCRA | hsa:171558 | K06056 |
| RBPJ | hsa:3516 | K06053 |
| RBPJL | hsa:11317 | K06053 |
| RFNG | hsa:5986 | K05948 |
| SNW1 | hsa:22938 | K06063 |

**Table S3: Prediction of functional impact of patient-derived gene mutations in NOTCH pathway components.**

| **Gene** | **Protein ID** | **Mutation** | **PPH2*** | **PROVEAN** | **MA** | **SIFT** | **Condel** | **SuSPect** |
| --- | --- | --- | --- | --- | --- | --- | --- | --- |
| DLL1 | O00548-1 | p.L264fs | n.p. | n.p. | n.p. | n.p. | n.p. | n.p. |
| DMXL2 | Q8TDJ6-1 | p.I2989V | - | - | + | ++ | - | - |
|  |  | p.T2810A | ++ | ++ | + | ++ | - | - |
|  |  | p.V586A | - | - | - | ++ | - | - |
| DTX1 | Q86Y01-1 | p.P138L | ++ | ++ | + | - | - | - |
| DTX3 | Q8N9I9-1 | p.P24T | - | - | - | ++ | - | - |
| DTX4 | Q9Y2E6-1 | p.V327F | ++ | ++ | + | ++ | - | - |
| DVL2 | O14641-1 | p.S170C | ++ | - | - | - | - | - |
| GXYLT2 | A0PJZ3-1 | p.H367P | ++ | ++ | + | ++ | - | - |
| HES5 | Q5TA89-1 | p.R31G | ++ | ++ | ++ | ++ | ++ | ++ |
| LFNG | Q8NES3-4 | p.I20N | - | - | n.p. | ++ | - | n.p. |
| NCSTN | Q92542-1 | p.L376P | ++ | ++ | + | ++ | ++ | - |
| NOTCH1 | P46531-1 | p.V1721L | ++ | - | + | - | ++ | + |
|  |  | p.A305S | - | - | - | - | - | + |
| NOTCH3 | Q9UM47-1 | p.C873S | ++ | ++ | ++ | ++ | ++ | ++ |
|  |  | p.E787K | - | - | - | - | - | - |
|  |  | p.Q731X | n.p. | n.p. | n.p. | n.p. | n.p. | n.p. |
| NOTCH4 | Q99466-1 | p.N1926K | - | - | - | ++ | - | - |
| NUMB | P49757-1 | p.P267A | ++ | ++ | + | - | - | + |

*PPH2: Polyphen-2, MA: MutationAssessor; mutations are evaluated to have a minor/negligible (-), medium (+) or high (++) impact or are not predictable with this tools (n.p.)

**Table S4: Somatic nucleotide variants and InDels of the TCGA-LIHC cohort (N=364).**

See Excel file attached

**Table S5: Pathway analysis of differentially regulated genes upon HES5wt expression.**

| **Name** | **ID** | **Gene count** | **Fold enrichment** | **p-value** | **Adj.**  **p-value** |
| --- | --- | --- | --- | --- | --- |
| Signaling pathways regulating pluripotency of stem cells | hsa04550 | 40 | 2.58 | 2.45E-08 | 6.83E-06 |
| Axon guidance | hsa04360 | 32 | 2.28 | 1.38E-05 | 0.002 |
| Rap1 signaling pathway | hsa04015 | 45 | 1.94 | 1.71E-05 | 0.002 |
| Pathways in cancer | hsa05200 | 70 | 1.61 | 4.72E-05 | 0.003 |
| Thyroid hormone signaling pathway | hsa04919 | 28 | 2.22 | 8.59E-05 | 0.005 |
| Ras signaling pathway | hsa04014 | 45 | 1.80 | 1.10E-04 | 0.005 |
| PI3K-Akt signaling pathway | hsa04151 | 61 | 1.60 | 1.96E-04 | 0.008 |
| Biosynthesis of unsaturated fatty acids | hsa01040 | 10 | 3.93 | 4.58E-04 | 0.016 |
| Glioma | hsa05214 | 18 | 2.50 | 5.11E-04 | 0.016 |
| Proteoglycans in cancer | hsa05205 | 39 | 1.76 | 5.27E-04 | 0.015 |

* adj. p-value by Benjamini-Hochberg procedure

**Table S6: List of top 20 genes bound by MYC in HepG2 cells (ENCODE ENCSR000DLR) and sorted by highest fold change of down regulation.**

| **GeneSymbol** | **Fold difference HES5wt +Dox vs. -Dox** | **-log10(p-value)** | **Adjusted p-value** |
| --- | --- | --- | --- |
| **HES1** | -2.722 | 14.982 | <0.001 |
| AHSG | -2.112 | 4.121 | 0.007 |
| RBP4 | -1.844 | 8.864 | <0.001 |
| **ODC1** | -1.805 | 13.871 | <0.001 |
| AGMAT | -1.793 | 10.795 | <0.001 |
| PDGFA | -1.727 | 14.423 | <0.001 |
| CCL20 | -1.706 | 7.086 | <0.001 |
| APOB | -1.703 | 7.678 | <0.001 |
| CEBPA | -1.578 | 6.347 | <0.001 |
| SMLR1 | -1.537 | 3.392 | 0.026 |
| ASGR1 | -1.473 | 7.348 | <0.001 |
| SLC7A2 | -1.449 | 6.431 | <0.001 |
| CP | -1.395 | 4.506 | 0.004 |
| NR0B2 | -1.369 | 9.939 | <0.001 |
| ZFP36L2 | -1.355 | 9.799 | <0.001 |
| BCOR | -1.309 | 17.520 | <0.001 |
| ANGPTL8 | -1.302 | 4.386 | 0.004 |
| **LDHA** | -1.252 | 12.961 | <0.001 |
| CGNL1 | -1.246 | 11.646 | <0.001 |
| EFNA1 | -1.202 | 6.017 | <0.001 |

**Table S7: Primers used for qRT-PCR.**

| **Gene** | **Accession** | **Sequence 5’-3’** | **Amplicon** |
| --- | --- | --- | --- |
| AFP | NM_001134.2 | Fwd: ACTGAATCCAGAACACTGCATAG | 128 bp |
|  |  | Rev: GCTTCTTGAACAAACTGGGCA |  |
| BIRC5 (Survivin) | NM_001168.3 | Fwd: TTTCTCAAGGACCACCGCATC | 126 bp |
|  |  | Rev: CAAGTCTGGCTCGTTCTCAG |  |
| CCND1 | NM_053056.2 | Fwd: GATCAAGTGTGACCCGGACTG | 101 bp |
|  |  | Rev: CCTTGGGGTCCATGTTCTGC |  |
| CDH2 | NM_001792.5 | Fwd: TGGCAGCTGGACTTGATCGAG | 128 bp |
|  |  | Rev: GACATCTGTCACTGTGATGACGG |  |
| CK19 | NM_002276.5 | Fwd: ACTACACGACCATCCAGGAC | 135 bp |
|  |  | Rev: GAGCCTGTTCCGTCTCAAAC |  |
| Ck19 (mouse) | NM_008471.3 | Fwd: TGGCTGTGTCTGATGGGCT | 244 bp |
|  |  | Rev: TTGGAGTTGTCAATGGTGGCA |  |
| CTGF (CCN2) | NM_001901.3 | Fwd: CCAAGGACCAAACCGTGG | 181 bp |
|  |  | Rev: CTGCAGGAGGCGTTGTCAT |  |
| CYR61 (CCN1) | NM_001554.5 | Fwd: AGCCTCGCATCCTATACAACC | 143 bp |
|  |  | Rev: TTCTTTCACAAGGCGGCACTC |  |
| FOXM1 | NM_202002.3 | Fwd: ATAGCAAGCGAGTCCGCATT | 284 bp |
|  |  | Rev: TTCCTCCCCAGGCTGGATTT |  |
| HES1 | NM_005524.4 | Fwd: AAGAAAGATAGCTCGCGGCA | 71 bp |
|  |  | Rev: CGGAGGTGCTTCACTGTCAT |  |
| HES5 | NM_001010926.4 | Fwd: CCGGTGGTGGAGAAGATG | 135 bp |
|  |  | Rev: GACAGCCATCTCCAGGATGT |  |
| HES5_CO | codon-optimized | Fwd: GCTCCTGCCAAAGAGCCTAA | 120 bp |
|  |  | Rev: TCACCAAGGCCTCCAAAGTC |  |
| HNF4A | NM_178849.2 | Fwd: CTCAAGAAATGCTTCCGGGC | 154 bp |
|  |  | Rev: GGGAGGTGATCTGTCGGGA |  |
| LDHA | NM_005566.4 | Fwd: CATGGCCTGTGCCATCAGTAT | 129 bp |
|  |  | Rev: GGTGTTCTAAGGAAAAGGCTGC |  |
| LGR5 | NM_003667.4 | Fwd: CCGCTTCCTGGAGGAGTTAC | 206 bp |
|  |  | Rev: AACAGCTTGGGGGCACATAG |  |
| MCAM | NM_006500.3 | Fwd: AAACATCCAGGTCAACCCCC | 184 bp |
|  |  | Rev: ACCACTCGACTCCACAGTCT |  |
| Mcam (mouse) | NM_023061.2 | Fwd: AATGTCGTGGGCATCCATGT | 209 bp |
|  |  | Rev: ACTAGGCGTGCACTCAGAAC |  |
| MCM2 | NM_004526.4 | Fwd: CATCAGCGACATGTGCAAAG | 46 bp |
|  |  | Rev: GTTCACCACCAGGCTCTCAC |  |
| NES | NM_006617.2 | Fwd: CAGCGTTGGAACAGAGGTTG | 120 bp |
|  |  | Rev: GAGCGATCTGGCTCTGTAGG |  |
| NOTCH1 | NM_017617.5 | Fwd: TGCAGAACAACAGGGAGGAG | 188 bp |
|  |  | Rev: CAGGTTGTACTCGTCCAGCA |  |
| ODC1 | NM_002539.3 | Fwd: AGTTGGTTTTGCGGATTGCC | 210 bp |
|  |  | Rev: CAAAAACACAGCGGGCATCA |  |
| PDGFA | NM_002607.5 | Fwd: GGCCGCGCTCCCTAAG | 232 bp |
|  |  | Rev: ATGCTGTGGATCTGACTGCG |  |
| SKIL | NM_005414.5 | Fwd: GATGTGTGTGGGGTTCGGA | 204 bp |
|  |  | Rev: GTCTGCTGGTCTGTGCTGAA |  |
| SOX2 | NM_003106.4 | Fwd: AACCAGCGCATGGACAGTTA | 180 bp |
|  |  | Rev: CGAGCTGGTCATGGAGTTGT |  |
| SOX4 | NM_003107.2 | Fwd: CCAAATCTTTTGGGGACTTTT | 65 bp |
|  |  | Rev: CTGGCCCCTCAACTCCTC |  |
| SRSF4 | NM_005626.4 | Fwd: TGCAGCTGGCAAGACCTAAA | 80 bp |
|  |  | Rev: TTTTTGCGTCCCTTGTGAGC |  |
| Srsf4 (mouse) | NM_020587.2 | Fwd: CGAGTCATTGTTGAGCACGC | 196 bp |
| Srsf4 (mouse) | NM_020587.2 | Fwd: CGAGTCATTGTTGAGCACGC | 196 bp |
|  |  | Rev: CTCCTGCCTGACGCATGTAA |  |
| TEAD4 | NM_003213.4 | Fwd: TGGAGTTCTCTGCCTTCCTG | 80 bp |
|  |  | Rev: GGACTGGCCAATGTGCACGA |  |
| VCAM1 | NM_001078.4 | Fwd: GGACCACATCTACGCTGACAA | 161 bp |
|  |  | Rev: CTCCAGAGGGCCACTCAAAT |  |
| VIM | NM_003380.5 | Fwd: GAAAGTGTGGCTGCCAAGAACC | 76 bp |
|  |  | Rev: CAGCCTCAGAGAGGTCAGCAA |  |
| Vim (mouse) | NM_011701.4 | Fwd: GGATCAGCTCACCAACGACA | 178 bp |
|  |  | Rev: AAGGTCAAGACGTGCCAGAG |  |

**Table S8: Overlapping primers used for site-directed mutagenesis.**

| Primer name | Primer sequences 5’-3’ |
| --- | --- |
| pDONR-HES5wt (human) | Fwd: ctgctgttgatgcggtcgcgccgcatc  Rev: gatgcggcgcgaccgcatcaacagcag |
| pDONR-HES5-S34D (human) | Fwd: cagttgctcgatgctgtcgttgatgcggtcgcgc  Rev: gcgcgaccgcatcaacgacagcatcgagcaactg |
| pDONR-HES5-S35D (human) | Fwd: gcttcagttgctcgatgtcgctgttgatgcggtcgc  Rev: gcgaccgcatcaacagcgacatcgagcaactgaagc |
| pT3-Hes5-R31G  (mouse) | Fwd: ctgctgttgatgccgtcccgacgcatc  Rev: gatgcgtcgggacggcatcaacagcag |

**Table S9: Primers used for ChIP qRT-PCR.**

| Primer name | Accession | Primer sequences 5’-3’ | Amplicon |
| --- | --- | --- | --- |
| HES1#1 | NM_005524.4 | Fwd: CTTTAACCGCAGTCGCCGAG  Rev: GGGATGCCTGACCGCACTTA | 70 bp |
| HES1#2 | NM_005524.4 | Fwd: CGGCCGGCTGATGTCAAA  Rev: CGTAGGCTTTAGGTTCTGCGG | 147 bp |
| HES1-3’UTR | NM_005524.4 | Fwd: TGCCAAAGATGTTTGAAAATGCTC  Rev: TCTGGAAGAATCAGTTCGAAGACA | 116 bp |
| LDHA#1 | NM_005566.4 | Fwd: GGAGGGCAGCACCTTACTTA  Rev: GAACCCACGTGTGAGTCGG | 140 bp |
| LDHA#2 | NM_005566.4 | Fwd: CCGCCGATTCCGGATCTCA  Rev: AGGCAGTTGGCTCTACCCG | 118 bp |
| ODC1#1 | NM_002539.3 | Fwd: TTGAGGCGCCGCTTCCT  Rev: GTGAAGACGGGGGCAGAAC | 85 bp |
| ODC1#2 | NM_002539.3 | Fwd: TTCTGATCAGGTAACAGCAACTG  Rev: TGACCAACAGGCATCATGGAA | 147 bp |
| Negative Control | - | Fwd: ATGGTTGCCACTGGGGATCT  Rev: TGCCAAAGCCTAGGGGAAGA | 174 bp |

**Table S10: Primary and secondary antibodies.**

| Antigen | Species | Product/Company | Experiments* |
| --- | --- | --- | --- |
| AKT | Rabbit | 9272/ Cell Signaling | WB |
| AKT | Mouse | 40D4/ 2920/ Cell Signaling | IF, PLA |
| phospho-AKT (Ser473) | Rabbit | D9E/ 4060/ Cell Signaling | WB |
| β-Actin | Mouse | 08691001/ MP Biomedicals | WB |
| β-Tubulin | Mouse | 556321/ BD Biosciences | WB |
| Cy3 AffiniPure anti-Rabbit IgG | Goat | 111-165-045/ Jackson ImmunoResearch | IF |
| Anti-FLAG M2 | Mouse | F1804/ Sigma-Aldrich | co-IP |
| HES1 | Rabbit | D6P2U/ 11988/ Cell Signaling | WB |
| HES5 | Rabbit | HPA065458/ Atlas Antibodies | WB, IF, PLA |
| HES5 | Rabbit | EPR15578/ ab194111/ abcam | co-IP, ChIP |
| HNF4α | Rabbit | C-19/ sc-6556/ Santa Cruz | IHC |
| IRDye 680LT anti-mouse IgG | Donkey | 926-68022/ LI-COR Biosciences | WB |
| IRDye 680LT anti-rabbit IgG | Donkey | 926-68023/ LI-COR Biosciences | WB |
| IRDye 800CW anti-rat IgG | Goat | 926-32219/ LI-COR Biosciences | WB |
| IRDye 800CW anti-rabbit IgG | Donkey | 926-32213/ LI-COR Biosciences | WB |
| Ki67 | Rabbit | ab15580/ abcam | IHC |
| LDHA | Rabbit | C4B5/ 3582/ Cell Signaling | WB |
| NOTCH1 | Rat | 5B5/ 3447/ Cell Signaling | WB |
| NOTCH3 | Rabbit | D11B8/ 5276/ Cell Signaling | WB |
| NUP62 | Mouse | G-8/ sc-166870/ Santa Cruz | WB |
| NUP98 | Mouse | C-5/ sc-74578/ Santa Cruz | WB |
| NUP153 | Rabbit | HPA027896/ Atlas Antibodies | WB |
| ODC1 (SLC25A21) | Rabbit | LS-B8566/ LS Biosciences | WB |
| PARP | Rabbit | 9542/ Cell Signaling | WB |
| panCK | Rabbit | Z0622/ Agilent (Dako) | IHC |
| phospho-YAP (Ser127) | Rabbit | 4911/ Cell Signaling | WB |

* WB, Western blot; IF, immunofluorescence; PLA, proximity ligation assay; co-IP, co-immunoprecipitation; ChIP, chromation immunoprecipitation; IHC, immunohistochemistry

**References**

1 Li H, Durbin R. Fast and accurate short read alignment with Burrows-Wheeler transform. *Bioinformatics* 2009; 25: 1754-1760.

2 Jones DT, Hutter B, Jager N, Korshunov A, Kool M, Warnatz HJ *et al*. Recurrent somatic alterations of FGFR1 and NTRK2 in pilocytic astrocytoma. *Nature genetics* 2013; 45: 927-932.

3 Jones DT, Jager N, Kool M, Zichner T, Hutter B, Sultan M *et al*. Dissecting the genomic complexity underlying medulloblastoma. *Nature* 2012; 488: 100-105.

4 Li H, Handsaker B, Wysoker A, Fennell T, Ruan J, Homer N *et al*. The Sequence Alignment/Map format and SAMtools. *Bioinformatics* 2009; 25: 2078-2079.

5 Stein LD, Knoppers BM, Campbell P, Getz G, Korbel JO. Data analysis: Create a cloud commons. *Nature* 2015; 523: 149-151.

6 Wang K, Li M, Hakonarson H. ANNOVAR: functional annotation of genetic variants from high-throughput sequencing data. *Nucleic acids research* 2010; 38: e164.

7 Rimmer A, Phan H, Mathieson I, Iqbal Z, Twigg SRF, Consortium WGS *et al*. Integrating mapping-, assembly- and haplotype-based approaches for calling variants in clinical sequencing applications. *Nature genetics* 2014; 46: 912-918.

8 Gonzalez-Perez A, Perez-Llamas C, Deu-Pons J, Tamborero D, Schroeder MP, Jene-Sanz A *et al*. IntOGen-mutations identifies cancer drivers across tumor types. *Nature methods* 2013; 10: 1081-1082.

9 Talevich E, Shain AH, Botton T, Bastian BC. CNVkit: Genome-Wide Copy Number Detection and Visualization from Targeted DNA Sequencing. *PLoS Comput Biol* 2016; 12: e1004873.

10 Kleinheinz K, Bludau I, Hübschmann D, Heinold M, Kensche P, Gu Z *et al*. ACEseq - allele specific copy number estimation from whole genome sequencing. *bioRxiv* 2017.

11 Cancer Genome Atlas Research Network. Electronic address wbe, Cancer Genome Atlas Research N. Comprehensive and Integrative Genomic Characterization of Hepatocellular Carcinoma. *Cell* 2017; 169: 1327-1341 e1323.

12 Sethi MK, Buettner FF, Krylov VB, Takeuchi H, Nifantiev NE, Haltiwanger RS *et al*. Identification of glycosyltransferase 8 family members as xylosyltransferases acting on O-glucosylated notch epidermal growth factor repeats. *The Journal of biological chemistry* 2010; 285: 1582-1586.

13 Sethi N, Yan Y, Quek D, Schupbach T, Kang Y. Rabconnectin-3 is a functional regulator of mammalian Notch signaling. *The Journal of biological chemistry* 2010; 285: 34757-34764.

14 Dai M, Wang P, Boyd AD, Kostov G, Athey B, Jones EG *et al*. Evolving gene/transcript definitions significantly alter the interpretation of GeneChip data. *Nucleic acids research* 2005; 33: e175.

15 Huang da W, Sherman BT, Lempicki RA. Systematic and integrative analysis of large gene lists using DAVID bioinformatics resources. *Nat Protoc* 2009; 4: 44-57.

16 Huang da W, Sherman BT, Lempicki RA. Bioinformatics enrichment tools: paths toward the comprehensive functional analysis of large gene lists. *Nucleic acids research* 2009; 37: 1-13.

17 Sommer C, Strähle, C, Köthe, U, Hamprecht, FA. ilastik: Interactive Learning and Segmentation Toolkit. *Eighth IEEE International Symposium on Biomedical Imaging (ISBI 2011)*, 2011.

18 Yu G, Wang LG, He QY. ChIPseeker: an R/Bioconductor package for ChIP peak annotation, comparison and visualization. *Bioinformatics* 2015; 31: 2382-2383.

19 Ploeger C, Waldburger N, Fraas A, Goeppert B, Pusch S, Breuhahn K *et al*. Chromosome 8p tumor suppressor genes SH2D4A and SORBS3 cooperate to inhibit interleukin-6 signaling in hepatocellular carcinoma. *Hepatology* 2016; 64: 828-842.

20 Schindelin J, Arganda-Carreras I, Frise E, Kaynig V, Longair M, Pietzsch T *et al*. Fiji: an open-source platform for biological-image analysis. *Nature methods* 2012; 9: 676-682.

21 Calvisi DF, Wang C, Ho C, Ladu S, Lee SA, Mattu S *et al*. Increased lipogenesis, induced by AKT-mTORC1-RPS6 signaling, promotes development of human hepatocellular carcinoma. *Gastroenterology* 2011; 140: 1071-1083.
